# Investigation of surface hardness, thermostability, tribo-corrosion, and microstructural morphological properties of microwave-synthesized high entropy alloy FeCoNiMnCu coating claddings on steel

**DOI:** 10.1038/s41598-024-55331-y

**Published:** 2024-03-02

**Authors:** Shubham Sharma, Shashi Prakash Dwivedi, Kahtan A. Mohammed, Abhinav Kumar, Fuad A. Awwad, M. Ijaz Khan, Emad A. A. Ismail

**Affiliations:** 1 Centre for Research Impact and Outcome, Chitkara University Institute of Engineering and Technology, Chitkara University, Rajpura, Punjab 140401 India; 2 School of Mechanical and Automotive Engineering, Qingdao University of Technology, Qingdao, 266520 China; 3 Department of Mechanical Engineering, Lebanese American University, Kraytem, Beirut, 1102-2801 Lebanon; 4 Department of Mechanical Engineering, Lloyd Institute of Engineering & Technology, Knowledge Park II, Greater Noida, Uttar Pradesh 201306 India; 5 Faculty of Pharmacy, Jabir Ibn Hayyan Medical University, Najaf, Iraq; 6 Department of Medical Physics, Hilla University College, Babylon, Iraq; 7 Department of Nuclear and Renewable Energy, Ural Federal University Named After the First President of Russia, Boris Yeltsin, 19 Mira Street, 620002 Ekaterinburg, Russia; 8 Department of Quantitative Analysis, College of Business Administration, King Saud University, P.O. Box 71115, 11587 Riyadh, Saudi Arabia; 9 Department of Mechanics and Engineering Science, Peking University, Beijing, 100871 China

**Keywords:** SOFC interconnects, Microwave energy, High entropy alloy, Cladding surface, Cladding layer, Engineering, Materials science, Physics

## Abstract

Deposition of high entropy alloy FeCoNiMnCu on SS-304 was carried out by microwave energy for application in “solid oxide fuel-cell (SOFC) interconnects”. The ball-milling has been performed by taking “Fe, Co, Ni, Mn, and Cu” in equal 20 wt. % of before deposited on SS-304 substrate. The deposited steel with 20% Fe 20% Co 20% Ni 20% Mn 20% Cu high entropy alloy (HEA) was exposed to thermal-exposure in the air for up to 10 weeks at 800 °C. The uniform cladding distribution of 20% Fe 20% Co 20% Ni 20% Mn 20% Cu HEA particles can be apparently observed on SS-304 substrate by utilizing Scanning Electron Microscope (SEM), and Optical microscopy analysis. Homogeneity in the interfacial layer was evident by employing Scanning Electron Microscope (SEM) characterization. Results have indicated that after the thermal exposure of deposited steel with 20% Fe 20% Co 20% Ni 20% Mn 20% Cu in the air for up to ten weeks at 800 °C, a “protective Cr_2_O_3_ layer”, and “high-entropy spinel coating” of (Fe, Co, Ni, Mn, Cu)_3_O_4_ have been formed. During microwave cladding, the emergence of harder-phases has contributed to the raised hardness. The wear behavior after coating of 20% Fe 20% Co 20% Ni 20% Mn 20% Cu HEA on SS-304 substrate has significantly enhanced due to the strengthened wear resistance and hardness of the coatings. Findings have exhibited that the formation of (Fe, Co, Ni, Mn, Cu)_3_O_4_ phase is a potential coating material for “SOFC interconnects” applications. Moreover, the cladding of SS304 with a composition of 20% Fe, 20% Co, 20% Ni, 20% Mn, and 20% Cu has demonstrated remarkable stability under thermal expansion studies. As the findings have revealed that the composite cladding has efficiently withstand significant variations in volume when subjected to elevated temperatures for a prolonged period of time, thus, exhibiting its superior thermal stability for SOFC-interconnect applications. Furthermore, the SEM images of the cladding surface, surface hardness, and tribocorrosion behavior of the coated material have been observed to identify the 20% Fe 20% Co 20% Ni 20% Mn 20% Cu HEA coating effect on SS-304 steel-substrate.

## Introduction

As a consequence of oxidising a fuel, SOFC is an electrochemical modification device that generates electricity. Fuel cells can be identified by their type of electrolyte material. Specifically, SOFCs has utilized a ceramic electrolyte. The SOFC interconnects are usually made of either a ceramic-layer or metallic that takes a seat among both individual cells. Its function is to fix every cell in series, as a result, the electricity generated by each cell can be combined. This category of fuel-cells has various benefits, including affordable pricing, minimal emissions, adaptability to different fuels, durability, effective power-output, and the ability to generate heat. The present study has attempted to fabricate SOFC interconnects by using SS-304 as a substrate with a coating of FeCoNiMnCu HEA^1^.

The demand of Composition- alloy complex (CCA) or HEAs has increased in recent years. There are one or two major solvents in the design or fabrication of traditional alloys. Whereas HEAs require five or more solvents to be fabricated or designed. However, while preparing HEAs, it must be kept in mind that the molar concentration of all the solvents is approximately equal. The HEAs have remarkable characteristics, for instance, prominent mechanical characteristics, resistance to wear, and resistance to corrosion^2,3^. There are various excellent characteristics of HEAs have been identified, such as TiZrNbWMo and CoCrFeNiTiAl alloy has showed high thermostability, CoCrFeNb HEA has reported high corrosion resistance, and CoCrNi-based medium entropy alloy has depicted the excellent comprehensive mechanical characteristics^1^.

Stainless steel is used in commercial or domestic products because of its good corrosion resistance even if the steel has slightly lower hardness property than iron and furthermore has revealed a lower wear resistance^2^. However, steel surface property may be increased by using cladding which is an excellent recent technology^3^. This is the latest technology in which impurity is added to the steel surface by using microwave technology and after which epitaxial growth is achieved on the steel surface. During the cladding process, the substrate undergoes partial dilution and which leads to produce a metallurgical bond. However, the new technology named laser beam cladding is widely practiced as a surface engineering technique even if currently microwave cladding is extensively used as surface engineering technology^4^.

However, there is a difference between Surface coating technologies and microwave techniques because Surface coating technologies include mainly magnetron sputtering, thermal spraying, plasma cladding, and laser cladding but for welding purposes, the microwave technique is used as a traditional materials processing^5^. The point of observation for Microwave welding is that it is a volumetric heating process that is used for achieves good binding with different materials^6,7^. During this process, the heating source pointed towards area-specific, internal, or external joints, and this heating specimen has absorbing properties at different dielectric conditions^8,9^. The microwave process was used to analyze its properties for heating any materials with respect to time, frequency, and average applied power. The magnetron device which is a conventional tube was used for energy development^10–12^. The maximum power value is 950 watts at 2.45 GHz frequency which was developed by a “single magnetron tube” in a closed cabinet. Due to the constant heating process in cladding, the technical characteristics of the material are maintained under a “microwave-source”^13–15^.

The available literature has suggested that there is a dearth of research concerning the microwave cladding of SS-304 employing a combination of the HEA utilising the ceramic coatings^16–18^. This research has endeavored to address this deficiency by investigating the evolution of cladding surfaces and their performance functional characteristics. In addition, the primary intent for this study is the synthesis or formation, and analysis for a microwave-cladded coatings on SS-304, which is composed of the Fe20Co20Ni20Mn20Cu20 HEA. To strengthen the characteristics of SS-304 steel, a material that is extensively employed owing to its favourable resistance to corrosion regardless its comparatively low hardness, in order to optimally fit its application under severe harsh environments.

The research has aimed to utilise the microwave energy technique produced HEAs for examining the formation of SS-304 cladding from a blend-combination comprising the blend-combination of 20%Fe, 20%Co, 20%Ni, 20%Mn, and 20%Cu. The microstructural analysis has entailed the examination of the cladding surface in order to gain insights into the material's composition, homogeneity, and potential imperfections. The influence caused by cladding on mechanical characteristics, like surface hardness has been evaluated in order to ascertain how the material's hardness is rising. The durability of the cladded material has been assessed under harsh environments, thereby, it is necessary to assess its corrosion resistance both prior to and subsequent to thermal exposure at 800 °C. The cladded samples' structural changes subsequent to thermal exposure has been examined with specific emphasis on the development of protecting shielding coating layers. In addition, the objectives of this research have been aimed at addressing concerns such as the implications of cladding on micro structure, surface hardness, as well as the corrosion performance; the development of barrier layers, shielding layers, barriers to corrosion or protective layers throughout thermal exposure; and the resulting ensuing modification in the substrate's characteristics during the method of deposition. Moreover, the intention for this study is to enhance the effectiveness in the microwave cladding technique by optimising numerous parameters including temperature, content of metallic-powder, environmental conditions, duration of irradiation and pre-heating time-period, separators and susceptors.

As far as the novelty for the present investigation is concerned or this study has actually distinguished from the already existing literary-studies by thoroughly examining the utilisation of microwave cladding, which involves the application of a Fe20Co20Ni20Mn20Cu20 HEA to SS-304 steel through a volumetric heating technique which in turn leads to precisely control the mechanism of Fe20Co20Ni20Mn20Cu20 HEA deposition, thus, assuring homogeneity and uniformity cladding process. The utilisation of microwave cladding technique to deposit the Fe20Co20Ni20Mn20Cu20 HEA onto SS-304 constitutes the scientific novelty of this investigation. The formation of a protective shield layer of coatings with prospective applications for wear and corrosion resistance is facilitated by the microwave cladding method and the unique blend combination of elements (Fe, Co, Ni, Mn, Cu).

In addition, the primarily emphasis of this study is the utilisation of microwave cladding as a means to the raising the chemical as well as thermal stability at elevated temperatures, strengthening the resistance to wear and corrosion by forming protective coating shielding inhibitory layer of chromium oxide and spinel oxides, producing a uniformly homogenous cladding-layer, and enhancing the surface characteristics of SS-304 steel. The prospective enhancements in resistance to corrosion, hardness, stability, and structural-integrity are of crucial significance as this work has significantly contributed to the ameliorate the long-lasting reliability, durability, stability, and resilience. Moreover, the development of barrier-layers with protective coatings or protective shielding layers through the application of microwave cladding employing the Fe20Co20Ni20Mn20Cu20 HEA composition. The development of a protective Cr_2_O_3_-layer and a high-entropy spinel-coating (Fe, Co, Ni, Mn, Cu)_3_O_4_ represents an innovative advancement and novelty. Moreover, the observed enhancement in hardness can be ascribed to the development of harder-phases subsequent to microwave cladding, including FeNi_3_, Al_2_O_3_, NiCO, CuO, and Mn_2_O_3_. A substantial rise in hardness is caused by the incorporation of these phases during the cladding method that covers the novelty of this study.

Through providing route-paths for electric-current to flow as well as separation of both the fuel and oxidant-gases, SOFC interconnects have been served for connecting individual fuel-cells in a stack pile array of fuel-cells. In the challenging environment associated with an SOFC, the interconnects must demonstrate the superior thermo-stability, as well as resistance to corrosion. In comparison with prior literary studies, the intent of this work has to strengthened the characteristics of the interconnects through the utilisation of a microwave energy-assisted cladding method for depositing a Fe20Co20Ni20Mn20Cu20 HEA to SS-304 steel^19,20^. The Fe20Co20Ni20Mn20Cu20 HEA coating has been developed to strengthen the interconnects' thermostability, and resistance to corrosion. In addition, the developed coatings for SOFC Interconnects have contributed to enhanced thermostability at elevated temperatures. The catalytic-activity for electro-chemical methods has been strengthened in this manner. An enhanced resistance to corrosion as well as reduced resistance to contact is an additional application of developed coatings for SOFC interconnects. Furthermore, the SOFC reliable performance, and long term dependability has been attained for effective operational circumstances. Thus, available literature has portrayed that very less researchers have utilised a blend combination of Fe20Co20Ni20Mn20Cu20 HEA in the “surface-cladding of SS-304” by “microwave-energy” for the application in SOFC interconnects^16–18^. Hence, the study attempted to investigate the development of the “cladding surface of SS-304” by utilizing the mixture of “high entropy alloy FeCoNiMnCu by microwave-energy” and also observed microstructure, surface hardness, corrosion, and structural behavior of the cladding-surface.

## Materials and methods

### Base material

**Figure 1:**
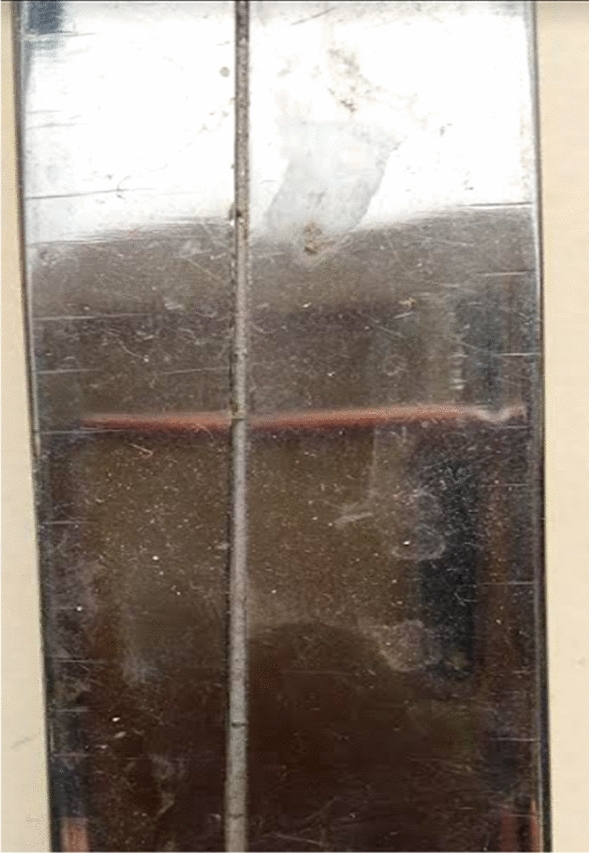
Photograph of SS 304.

**Figure 2:**
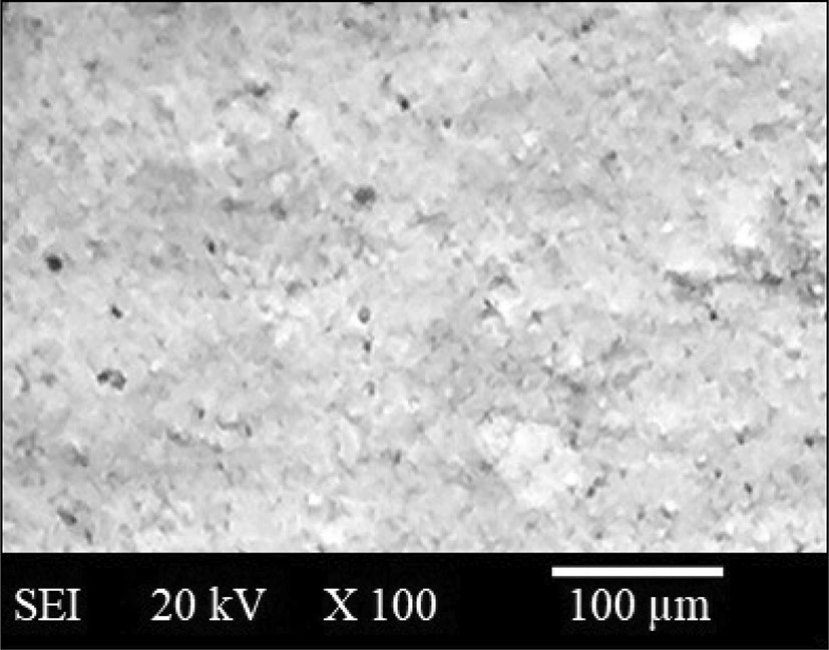
SEM image of SS-304.

Stainless steel 304 also known as the chromium-Nickel austenitic alloy is steel austenitic of a T 300 Series as depicted in the Fig. 1. It has a minimum of 0.08% carbon, 8% nickel, and 18% chromium. The SEM morphology for SS 304 has been exhibited in the Fig. 2. Some characteristics of chromium-Nickel austenitic alloy (Stainless steel 304) are the characteristics of appearance, ease of fabrication, ease of cleaning, remarkable trait in hardening by cold working, excellent toughness, deep drawing quality, corrosion/oxidation resistance, Forming and welding properties, etc.^4^. The measured hardness of SS-304 was 210 HV. The characteristics of SS-304 observed up to 0.16 m diameter/thickness have been exhibited in Table 1.

**Table 1:** Characteristics of SS-304-base material.

S. No	Characteristics	Values
1	“Vickers-hardness” (HV)	210
2	“Tensile Strength” (MPa)	610
3	“Density” (gm/cm^3^)	7.5
4	“Melting temperature” (℃)	1445

### Cladding particles

**Figure 3:**
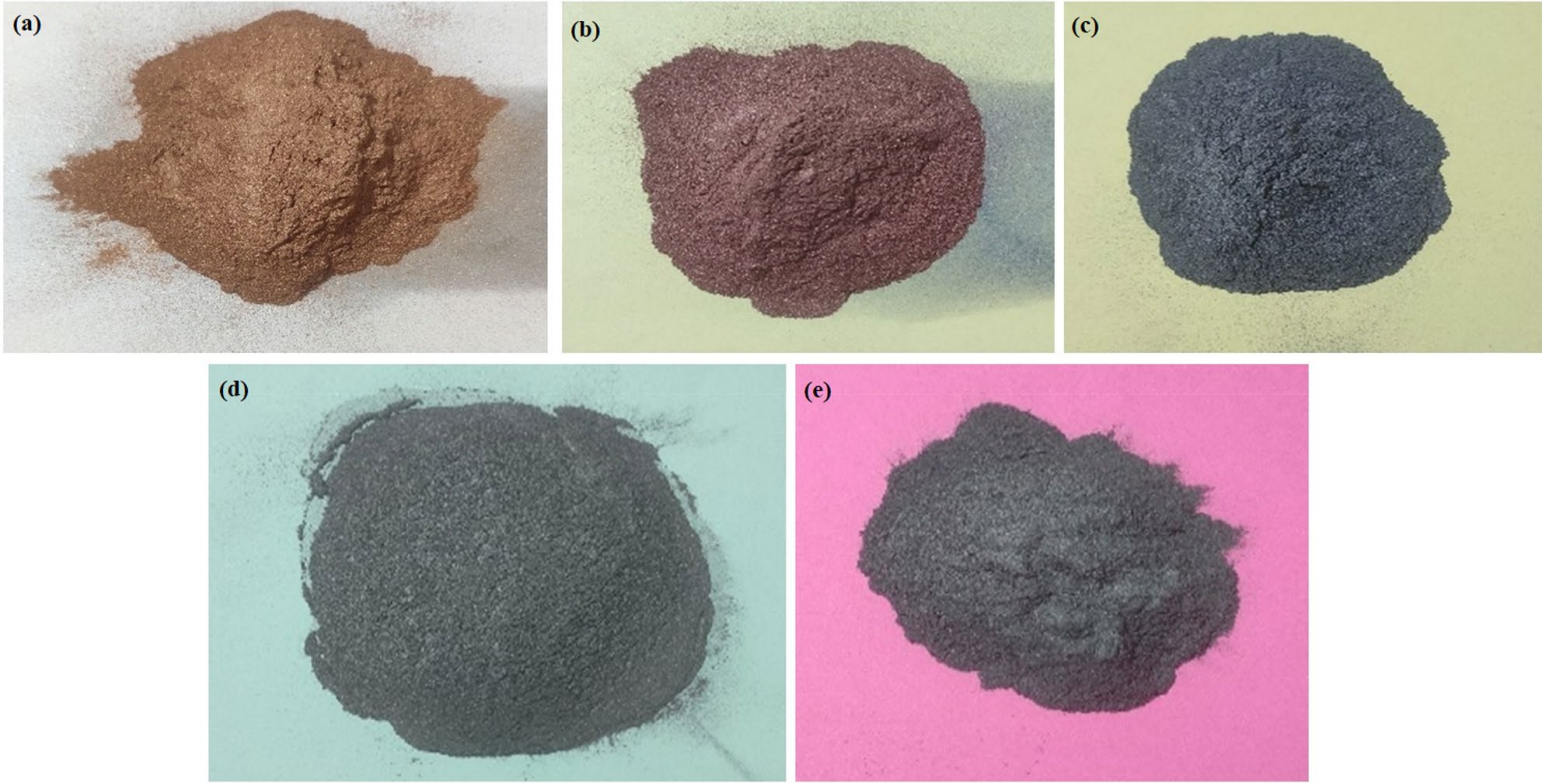
Photographs of (**a**) Cu, (**b**) Mn, (**c**) Ni, (**d**) Co, (**e**) Fe particulates.

**Figure 4:**
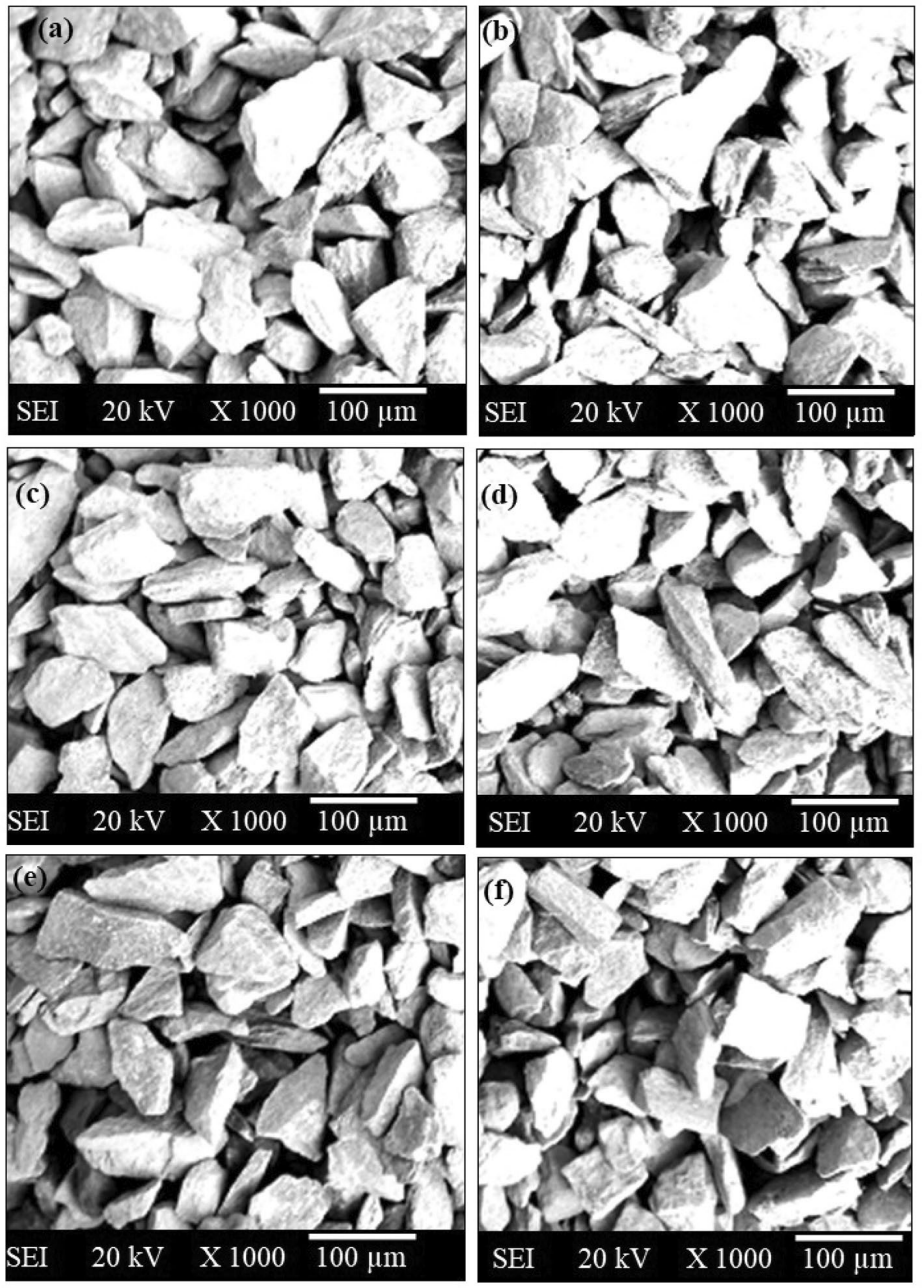
Powder SEM image of (**a**) Cu, (**b**) Mn, (**c**) Ni, (**d**) Co, (**e**) Fe, (**f**) the mixture of ball-milled 20% Fe 20% Co 20% Ni 20% Mn 20% Cu.

**Figure 5:**
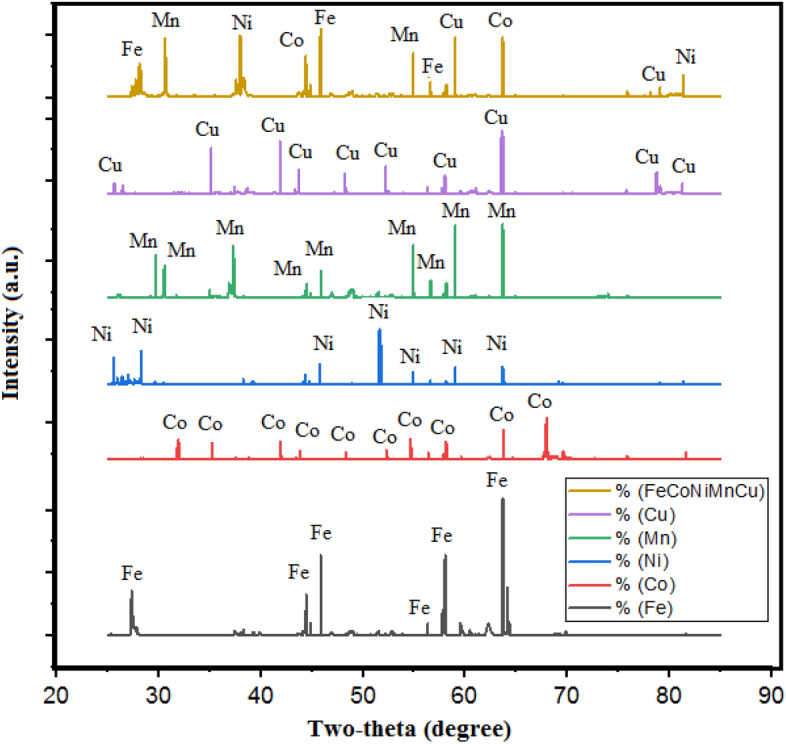
Powder XRD of Cu, Mn, Ni, Co, Fe, the mixture of 20% Fe 20% Co 20% Ni 20% Mn 20% Cu.

In this study, five different metal powders (Fe, Co, Ni, Mn, and Cu) have been chosen for the cladding on SS-304 steel as depicted in the Fig. 3. The Fe, Co, Ni, Mn, and Cu particles were selected as entropy alloys in powder form with an avg. particle size of 19 µm, 20 µm, 21 µm, 23 µm, and 24 µm as exhibited in the SEM examinations in Fig. 4. The entire five entropy alloys in the powder form were ball-milled. Equal weight percent (20%) have been taken during the mechanical alloying. Horizontal ball-milling has been used for mechanical alloying of HEA. The ball milling speed and BPR have been fixed as 200 rpm and 5:1 ratio respectively. The avg. particle size of ball-milled cladding-particulates is 17 µm. Powder XRD of individual powder and after the mechanical alloying of entropy alloy is shown in Fig. 5. An individual metal powders can be recognized by their distinctive diffraction peaks. Every metal has exhibited its own distinctive characteristic peaks. Greater crystallinity is indicated by diffraction peaks with greater intensity and sharpness in XRD analysis^16,21,22^. Diffraction peak width can also reveal particulars about the dimensions of crystalline domains. Narrow peaks imply larger crystalline domains. The blend's XRD pattern has unveiled that diffraction peaks are equivalent to the crystalline phases of Fe, Co, Ni, Mn, and Cu cladding-particulates. The proportion of each metal's crystalline phase in the blend can be determined from the relative integrated intensities of the peaks.

### Development of cladding

**Figure 6:**
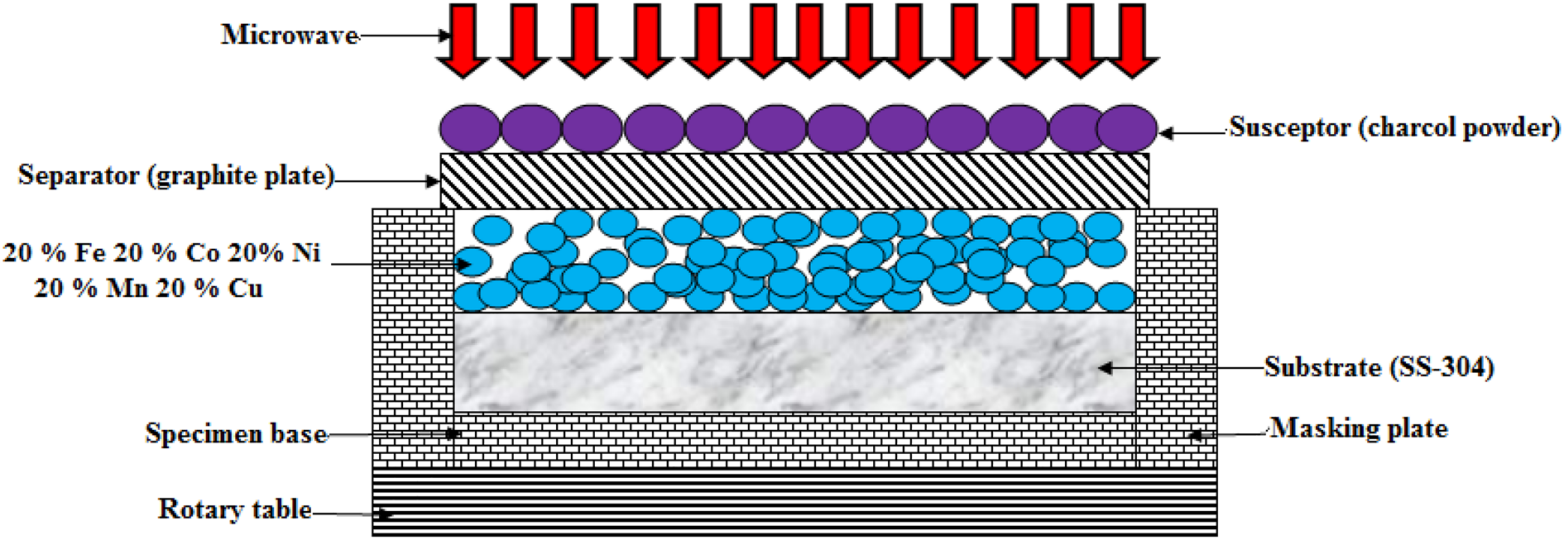
Schematic drawing of the experimental procedure.

**Figure 7:**
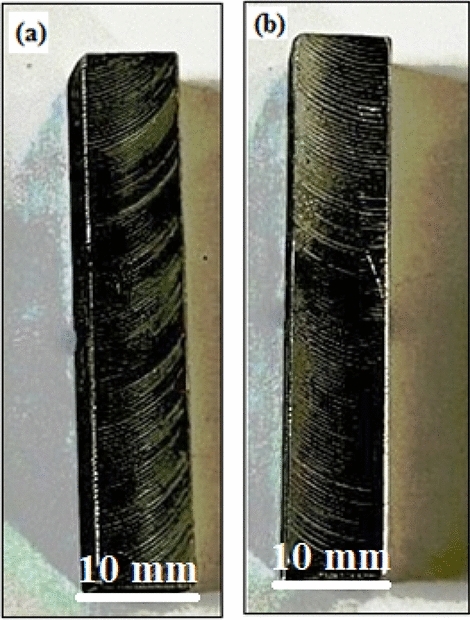
(**a**,**b**) Images of 20% Fe 20% Co 20% Ni 20% Mn 20% Cu on SS-304; (**a**) before thermal exposure in the air for up to 10 weeks at 800 °C, (**b**) after thermal exposure in the air for up to 10 weeks at 800 °C.

The current research involved the use of an “ultrasonic bath and alcohol” to clean the substrate (SS-304) before deposition. The mixture of 20% Fe 20% Co 20% Ni 20% Mn 20% Cu after ball-milling was preheated at 100℃ for 24 h. Preheating of the mixture of 20% Fe 20% Co 20% Ni 20% Mn 20% Cu after ball-milling particles were done. The powder employed in this study was preheated to remove moisture and then uniformly placed on an SS-304 substrate^23,24^. “Microwave hybrid heating (MHH)” was then conducted using a “multimode microwave applicator” at 900 W and a frequency of 2.45 GHz (Fig. 6). A homogenously-dispersion of the “preheated powder” was achieved before it was deposited on the SS-304 substrate. In order to prevent direct-contact of the particulates with “microwave radiation” at room-temp., the skin-depth of the predominant component of the hard-facing powder was set to approximately 4.5 µm at 2.45 GHz. For the purpose of overcoming the reflection of microwaves caused by the blend combination of 20% Fe 20% Co 20% Ni 20% Mn 20% Cu after ball-milling, through "microwave hybrid heating" (MHH), the "charcoal" was being employed as the "susceptor material" for clads as depicted in Fig. 6. According to Table 2, the optimal experimentation parameters for the preparation of microwave-cladded samples may be attained by employing the mixture of 20% Fe 20% Co 20% Ni 20% Mn 20% Cu after ball-milling on SS-304. The “microwave-coated steels” were subjected to “thermal-exposure” in the air for up to ten-weeks at 800 °C. Figure 7a,b exhibits the images (scale bar of 10 mm) of the “microwave-clad samples” before and after thermal exposure in the air for up to 10 weeks at 800 °C developed by using the mixture of 20% Fe 20% Co 20% Ni 20% Mn 20% Cu on SS-304 respectively. Table 3 has depicted the cladding compositions of 20% Fe 20% Co 20% Ni 20% Mn 20% Cu powder on SS-304 (Substrate).

**Table 2:** Prerequisites for conducting investigations for microwave-clad samples.

S. no	Parameters	Descriptions
1	Atmospheric-conditions	Ambient
2	Avg. duration for cooling	695 s
3	Irradiation duration	115 s
4	Pre-heating duration for metallic-powder	24 h
5	Temp. at which metallic-powder is pre-heated	120 °C
6	Metallic-powder	20% Fe 20% Co 20% Ni 20% Mn 20% Cu after ball-milling
7	Separators employed	Graphite-plate
8	Susceptors employed	Charcoal
9	Frequency	2.45 GHz
10	Power	900 W
11	Substrate	SS-304

**Table 3:** Coating Composition.

Sample No	Substrate	Coating of Fe (wt%)	Coating of Co (wt%)	Coating of Ni (wt%)	Coating of Mn (wt%	Coating of Cu (wt%)	Coating compositions
1	SS-304	20%	20%	20%	20%	20%	20%Fe20%Co20%Ni20%Mn20%Cu
2	SS-304	40%	15%	15%	15%	15%	40%Fe15%Co15%Ni15%Mn15%Cu
3	SS-304	15%	40%	15%	15%	15%	15%Fe40%Co15%Ni15%Mn15%Cu
4	SS-304	15%	15%	40%	15%	15%	15%Fe15%Co40%Ni15%Mn15%Cu
5	SS-304	15%	15%	15%	40%	15%	15%Fe15%Co15%Ni40%Mn15%Cu
6	SS-304	15%	15%	15%	15%	40%	15%Fe15%Co15%Ni15%Mn40%Cu

### Coating materials testing and equipment’s

In order to carry out the hardness measurements, XRD assessment, and microstructure analysis on cladding samples of SS 304, it is necessary to employ a testing equipment’s or apparatus that possesses the subsequent characteristics^25,26^:

#### SEM analysis

##### Type

FESEM analysis has been employed for higher-resolution imaging.

##### Detector

In this study, “secondary electron” (SE), as well as “back-scattered electron” (BSE) detectors have been employed for compositional contrast as well as surface imaging, respectively^25,26^.

##### Sample-stage

A motorized-stage is employed to attain accurate sample-orientation, placement, and positioning.

##### Chamber

To mitigate electron scattering, a vacuum-environment was being employed within the chamber.

For SEM imaging of SS-304 alloy with 20% Fe 20% Co 20% Ni 20% Mn 20% Cu coatings, used etchant was a mixture of 10% oxalic acid and 90% distilled water. This etchant is effective in revealing microstructural details and enhancing the contrast between different phases, allowing for a detailed examination of the sample's surface morphology.

#### XRD analysis

##### XRD type

For phase analysis, a “powdered X-ray diffractometer” was being employed.

##### X-ray source

A mono-chromatic, higher-intensity X-ray source, such as “Cu Kα radiation”.

##### Detector

A sensitive, higher-resolution device, such as a “scintillation detector”.

##### Goniometer

A “motorized-goniometer” is utilised to ensure accurate sample placement.

##### Sample holder

A sample-holder is an apparatus designed for holding cladding specimens and facilitates rotation.

#### Hardness examination equipment

##### Hardness-tester

**Figure 8:**
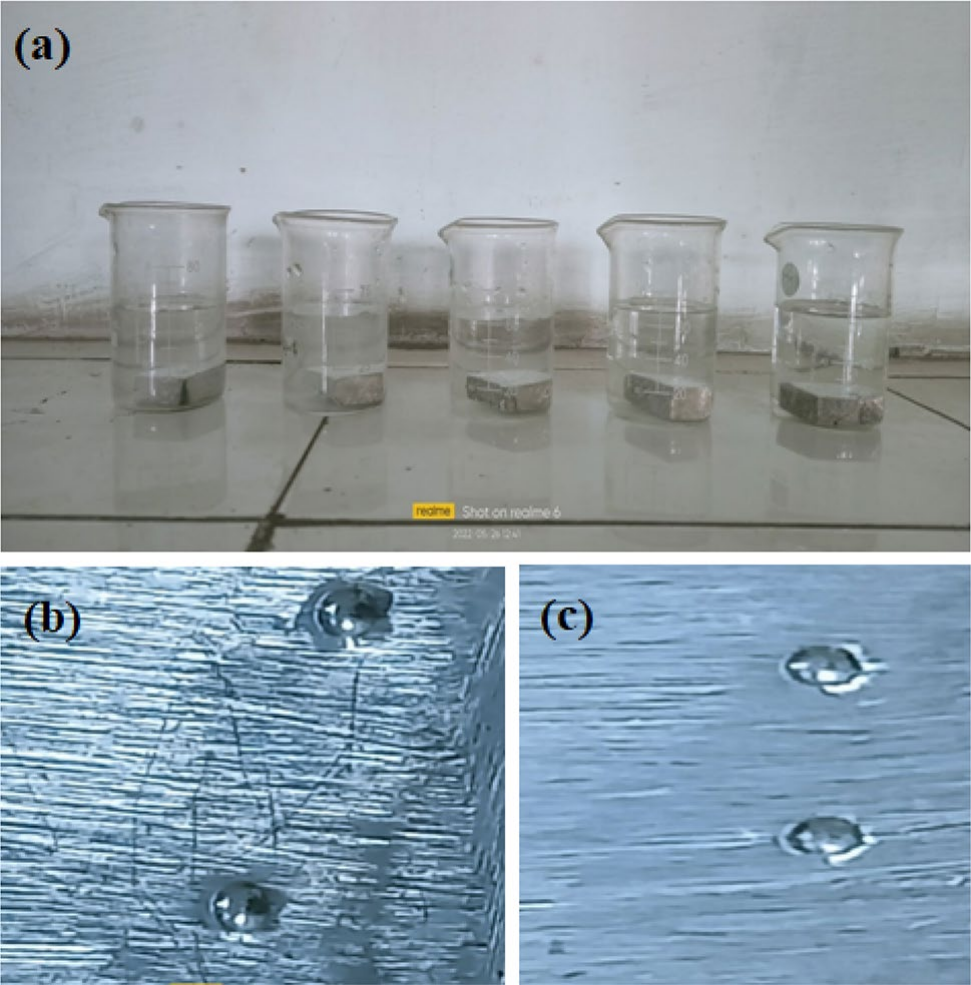
(**a**) Samples with Corrosion testing, (**b**,**c**) Hardness testing indentation.

The Vickers hardness tester has been employed to measures hardness on a designed scale utilising ASTM E384. The hardness tested specimen has been exhibited in Fig. 8b,c.

##### Load applications

A motorized loading application-based system that ensures consistent as well as precise loadings.

### Corrosion testing

The corrosion testing of SS-304 alloy samples with 20% Fe 20% Co 20% Ni 20% Mn 20% Cu coatings have involved an immersion in a 3.5 wt% NaCl media solution for 120 h (Fig. 8a) employing ASTM G44 standards. The experimental procedure has included an assessing resistance to corrosion, measuring corrosion loss, and analyzing surface morphology. This method has aided in evaluating the protective efficacy of diverse coatings against corrosive environments.

### Wear testing evaluation

**Figure 9:**
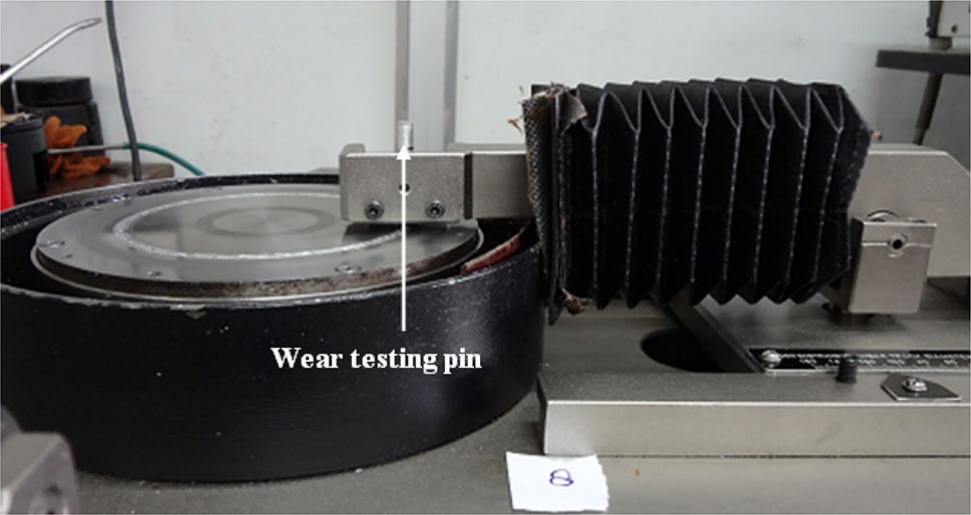
Pin on Disc Set-up with wear testing pin.

The standard method for conducting wear testing with the ASTM G 99 standard employing a pin-on-disc (POD) apparatus (as depicted in Fig. 9) on SS 304 steel that has been coated with 20% Fe 20% Co 20% Ni 20% Mn 20% Cu particles under specified circumstances, i.e., a sliding speed of 2 m/s, a sliding distance of 1000 m, and axial stresses of 15N, and the same has been elucidated as follows^25,26^:

i. *Specimen preparation:* The SS-304 specimen was prepared with a 20% Fe 20% Co 20% Ni 20% Mn 20% Cu coating.
ii. *POD set-up configuration:* The POD testing equipment, comprising a rotating-disc as well as a stationary-pin (counter-part), was assembled. The disc was constructed from a material that represented the state or conditions of wear.
iii. *Speed and load setup-related configurations:* This pin has been subjected to a pre-determined specific axial-load of 15N. The disc's speed of rotation was set up at 2 m per second.
iv. *Wear test trial-runs:* This rotating-disc was positioned in contact with the coated SS 304 specimen (pin). The POD apparatus was operated for a specified duration and sliding-distance of 1000 m.
v. *Data collection:* The wear-rate was determined by measuring as well as recording the weight-loss of the pin (coated SS304 specimen) both prior to and subsequent to the test.
vi. *Friction measurement:* In order to evaluate the tribological characteristics of the material, the coefficient of friction was being observed, measured, and recorded throughout the experiments.

## Rationale behind the selection of proposed material combination with 20% wt. percent during the mechanical alloying with scientific mechanism

In comparison from the extensive in-depth literature-study findings, the rationale motivation for examining the selected wt% (20%) as formulated blend combination during mechanical-alloying have been elucidated as follows:

The selection of an equivalent wt% (20%) for the mechanical alloying of Fe, Co, Ni, Mn, and Cu within the scope of the research is plausibly determined by numerous vital considerations and significant parameters that are crucial to accomplishing the desired intended characteristics in the Fe20Co20Ni20Mn20Cu20 HEA coatings as reported from the exhaustive literary-studies^25–28^. The fallowing subsequent factors have contributed to a rationale for the selection of the comparable percentage and 20%, firstly, the uniformity of the alloy's composition is achieved by assigning an equivalent weight percentage to each element. Compositional variation may have the profound noticeable implications on the characteristics of a Fe20Co20Ni20Mn20Cu20 HEA, making this a crucial step for developing a clearly defined and uniformly consistent alloy.

Secondly, an equi-atomic or nearly equi-atomic composition of numerous elements constitutes the basis of the Fe20Co20Ni20Mn20Cu20 HEA. The structural-integrity and mechanical characteristics of the alloy are considered to be enhanced by this blend-combination. By keeping a comparable percentage of each element in the alloy, the desirable equiatomic composition has been accomplished. Thirdly, the development of a uniform solid solution during the process of mechanical alloying is facilitated by the use of equivalent weight percentages. Ensuring homogenous morphology and characteristics throughout the coating is of paramount significance. It prevents the development of undesirable phases and promotes solid-solubility by ensuring that each element is uniformly distributed in the alloy matrix. Fourthly, the Enhanced Entropy, the expression "high entropy" signifies a significant degree of disorder or randomness within the arrangement of atoms. The utilisation of identical wt. percentages in an arrangement of various elements conduces to a rise in configurational entropy, which in turn induces disorder. The strengthened entropy has been reported to result in the development of unique characteristics, including enhanced resistance to corrosion, structural strength, durability, resilience, as well as mechanical strength. Then, the efficient alloying process, as the alloying method is made more efficient in the process of mechanical alloying through the utilisation of identical weight percentages. By implementing this process, the energy input throughout the milling process is uniformly, evenyl and homogenously dispersion between all elements, thereby inhibiting any preferred alloying of a specific element. As a consequence, the alloy composition becomes more consistent uniformly, reliable, and predictable.

Then, by inhibiting phase separation or precipitation-induced among distinct elements, alloy stability has been enhanced. Separation of phases as well as high-temperature exposure are considerably less probable to happen in an optimal composition, thereby enhancing the stability of the coating. Then, the utilisation of comparable wt% in the fabrication technique facilitates the control of numerous alloying elements, thus enhancing method of manufacturing. In regards to reliability and scalability, this simplicity may be advantageous. In addition, the alloying process is simplified and rendered more manageable and controllable by utilising an equal weight percentage. Due to the simplicity, convenience, and ease of experimental design and analysis, symmetrical contents, including 20% for every element, are usually recommended. Then, in order to attain a particular phase stability in the resultant Fe20Co20Ni20Mn20Cu20 HEA, the 20% choice for every constituent element might have been determined. Stable single-phase structures can result from selecting specific alloying compositions, such as the equiatomic composition (20% for each element). that was selected. Then, in order to explore the development of a high-entropy spinel-coating as well as an inhibitory shielding protective chromium-oxide layer, the coated material is afterwards subjected to 800 °C thermal conditions. A stable-alloy that is capable of withstanding as well as reacting effectively to such circumstances has been determined through identical weight-percentages. Moreover, air exposure to 800 °C for a maximum of ten weeks has been required for this study. In order to develop a barrier of protection, protective-film, shielding film-layer (Cr_2_O_3_ as well as (Fe, Co, Ni, Mn, Cu)_3_O_4_) that is capable of withstanding the aforementioned thermal environments, the selected composition along with weight percentage could be optimised. Then, the weight percentages of the elements are distributed equally, which promises an even balance-dispersion, and inhibits an alloy's properties from being heavily dominated by any particular element. To attain the intended characteristics and functionality efficacy, performance functionality, and effectiveness of the Fe20Co20Ni20Mn20Cu20 HEA, this balanced composition is essential. By maintaining a balanced composition, the occurrence of phase separation and the development of undesired intermetallic phases during the alloying method are significantly reduced. Then, the development of a complex crystalline-structure, which strengthens the thermomechanical, and resistance to corrosion, can be accomplished through the utilisation of multiple elements in equivalent proportions. The uniform distribution of elements may improve the overall performance of the alloy. Then, mechanical alloying is simplified by the use of equal weight percentages, which facilitates the optimisation and control of alloying conditions. The aforementioned factors hold specific significance with regard to the milling speed of 200 rpm, and the ball-to-powder proportion of 5:1. Then, an alloy with a homogeneous microstructure is the result of equal wt% of elements, which decreases the probability of segregation of elements, or irregular distribution within alloy. Ensuring uniformity is critical in order to accomplish uniform consistent characteristics throughout every aspect of the coating. Then, the precise composition, consisting of equal or identical weight percentages has been customised for utilisation in SOFC interconnects. The alloy is effective and appropriate for applications in SOFC environments owing to the enhanced corrosion resistance caused by the protective barrier coating layers that form as an outcome of thermal exposure. Then, it may have been determined that an optimal blend combination for the particular application of corrosion resistance, wear resistance, and SOFC interconnects comprised every element with an equal weight percentage of twenty percent. This combination would have produced reasonable appropriate characteristics including hardness, resistance to corrosion, and thermostability.

In context with the prior literary-studies on the microwave-cladded synthesis coatings with their respective specific blend-combinations and the scholarly analysis have reported that a 20% wt. percentage of every constituent element is appropriate for the intended desired purpose as it has contributed to favourable characteristics in alloy-combinations that are identical or comparable^25–28^.

## Results and discussion

### Microstructure investigation

**Figure 10:**
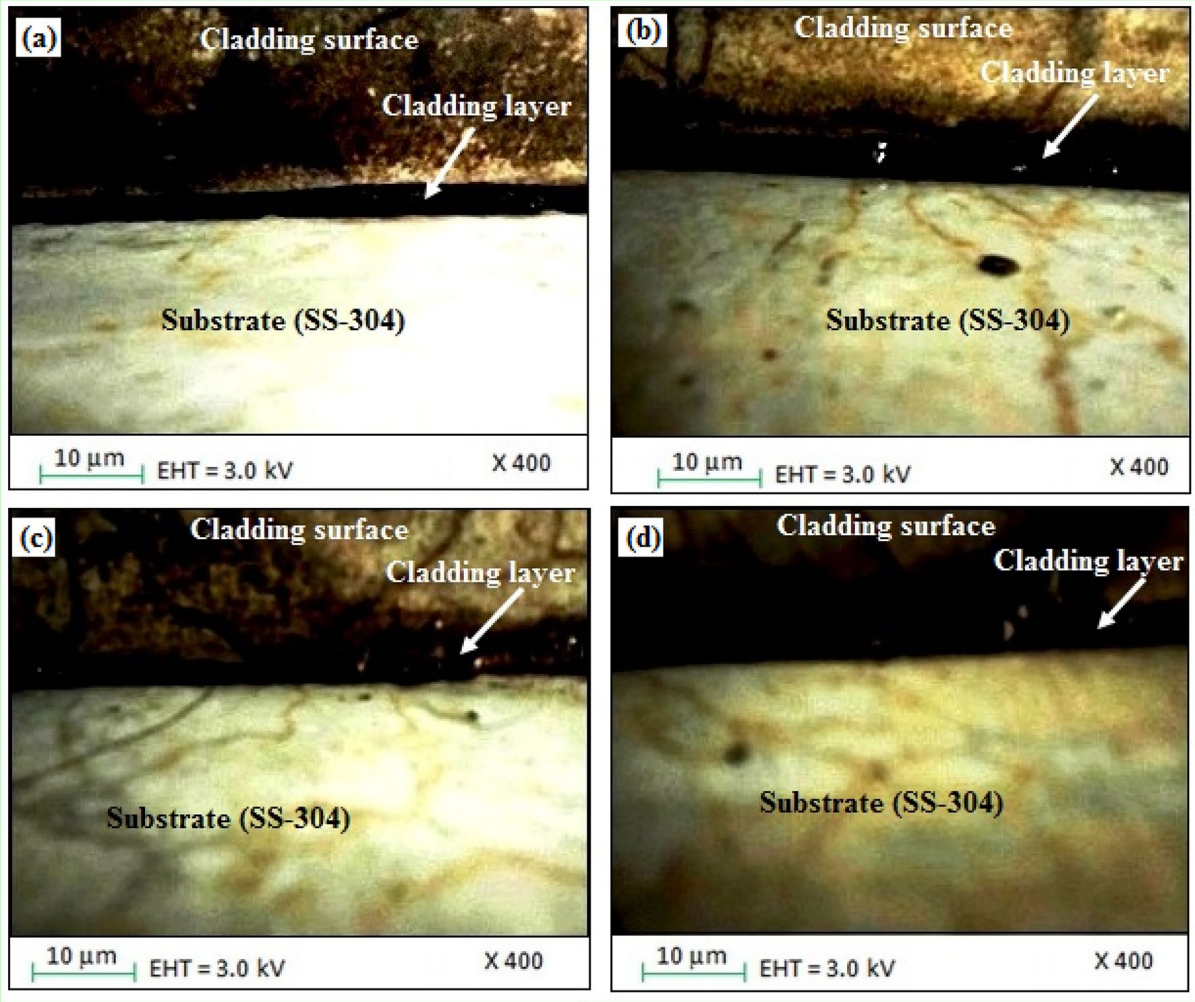
SEM images of the cladding surface and the cladding layer of SS-304 coating with Fe20Co20Ni20Mn20Cu20 HEA.

**Figure 11:**
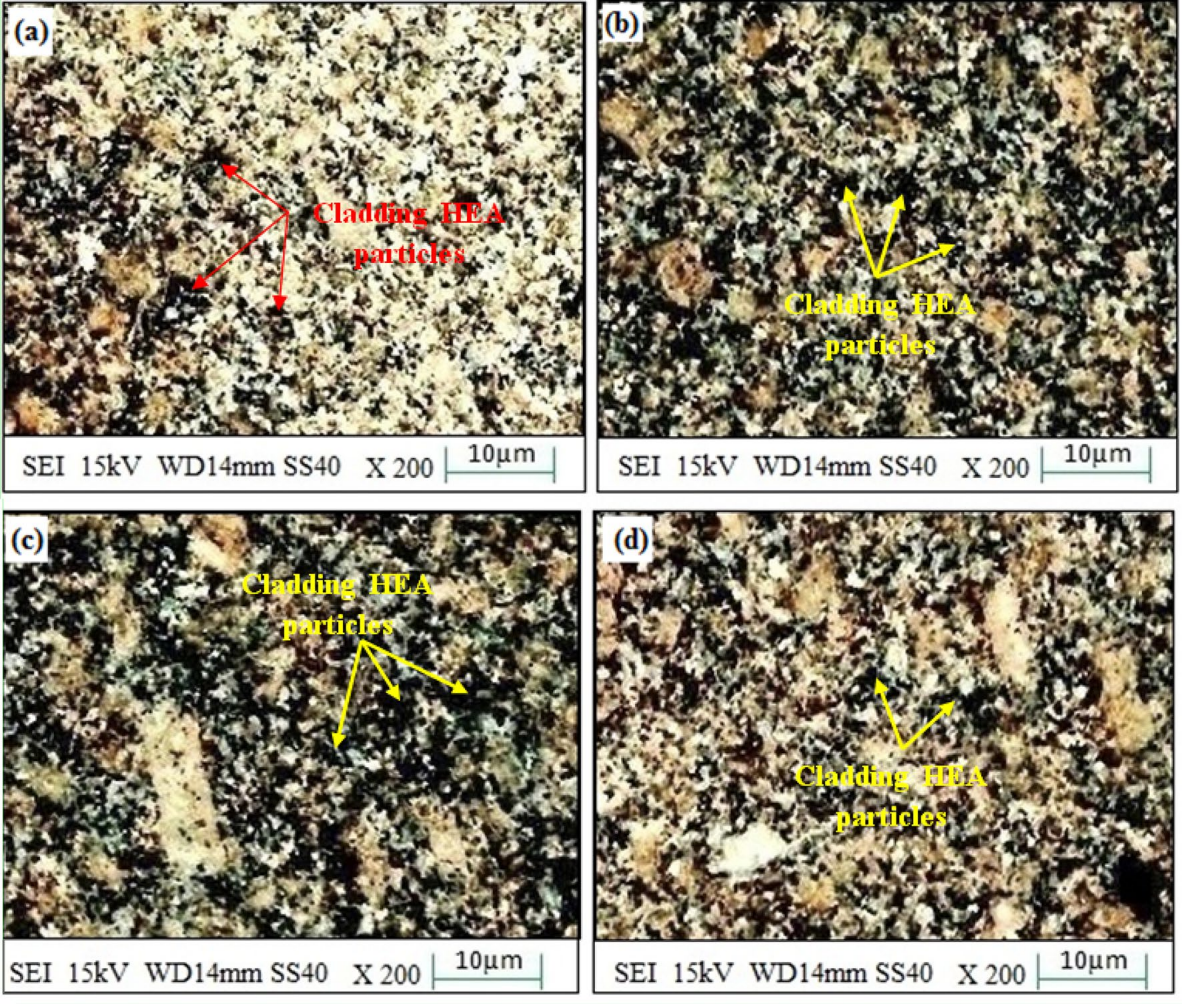
SEM images of SS-304 coatings with Fe20Co20Ni20Mn20Cu20 HEA before thermal exposure in the air for up to 10 weeks at 800 °C.

By keeping the microwave parameters at an optimum level, a uniform cladding layer was obtained, which resulted in fewer dark pixels and higher homogeneity, as shown in Fig. 10a-c. Dark image-elements in the SEM image could represent regions with maximum atomic number components or regions with distinct compositions or microstructures. They may be construed as concentrated areas of coating elements (Fe, Co, Ni, Mn, Cu) in accordance with the microwave cladding. A cladding layer with higher homogeneity likely has coating components distributed across it more evenly, which is necessary for persistent material characteristics. However, some cracks were observed between the cladding layer and substrate, as depicted in Fig. 10d. In comparison with the findings from the prior-studies, it has been discovered that at times, the micro-fractures amongst the cladding layer and the steel-substrate may be the result of variables like different cooling rates during the cladding process, residual stresses^29–31^. From the outcomes of existing-sources, the occurrence of stress as well as micro-fracture formation throughout the cladding technique can be attributed to differential cooling-rates^32,33^. Although, the cladding technique can give rise to residual-stresses, which have the potential to contribute to the formation of tiny-cracks as reported previously^34,35^. Another factor that can contribute to the development of micro-cracks is inadequate bonding among the cladding layer as well as substrate as unveiled by the findings from prior-literature^36,37^. Thereby, the developed coatings may be more susceptible to wear-related damage at these tiny-crack sites, which can result in lower wear resistance as reported by the scholarly studies^38,39^. Now, herein this work, a higher concentration of coating elements may be present in darker areas of the SEM images. Due to the presence of these harder elements, this might indicate areas with enhanced hardness. Figure 11 has depicted the SEM image of SS-304 coating with 20% Fe 20% Co 20% Ni 20% Mn 20% Cu before thermal exposure in the air for up to 10 weeks at 800 °C for the cladding surface. Following the "microwave-processing" (hot-deformation), the "microwave cladding sample coating” with 20% Fe 20% Co 20% Ni 20% Mn 20% Cu cooled, and its structure has transformed from higher-temp. austenite-phase to the phase of a lower-temperature, as a consequence. The structure of the coated sample altered as it cooled. The sample's microstructure, which was initially austenitic, changed as a result of the controlled cooling process to lower temperature phases. Because it has affected the material's behavior and characteristics, this transition is substantial. The intrinsic microstructures of the majority of ferritic-steels (those without the composition of 20% Fe, 20% Co, 20% Ni, 20% Mn, 20% Cu) undergo changes that result in the formation of pearlite and ferrite, nevertheless the microwave-cladded samples have displayed a distinct behavior. This study puts fewer significance on interlamellar-spacing and pearlite formation and instead concentrates on the ferrite grain-size. This is as a result of the fact that these factors only make a slight contribution to the composition's strength^25–28^. Following microwave processing, the solidification of the microwave-clad sample was examined, demonstrating the existence of distinguishable ferrite grains. Two crucial factors that are crucial in this situation are the “retained-strain”, and the desired “austenite grain-size”. The “cooling rate”, “composition”, and “deformation history” are instances of exterior factors that have an impact on both of these variables^40–42^.

**Figure 12:**
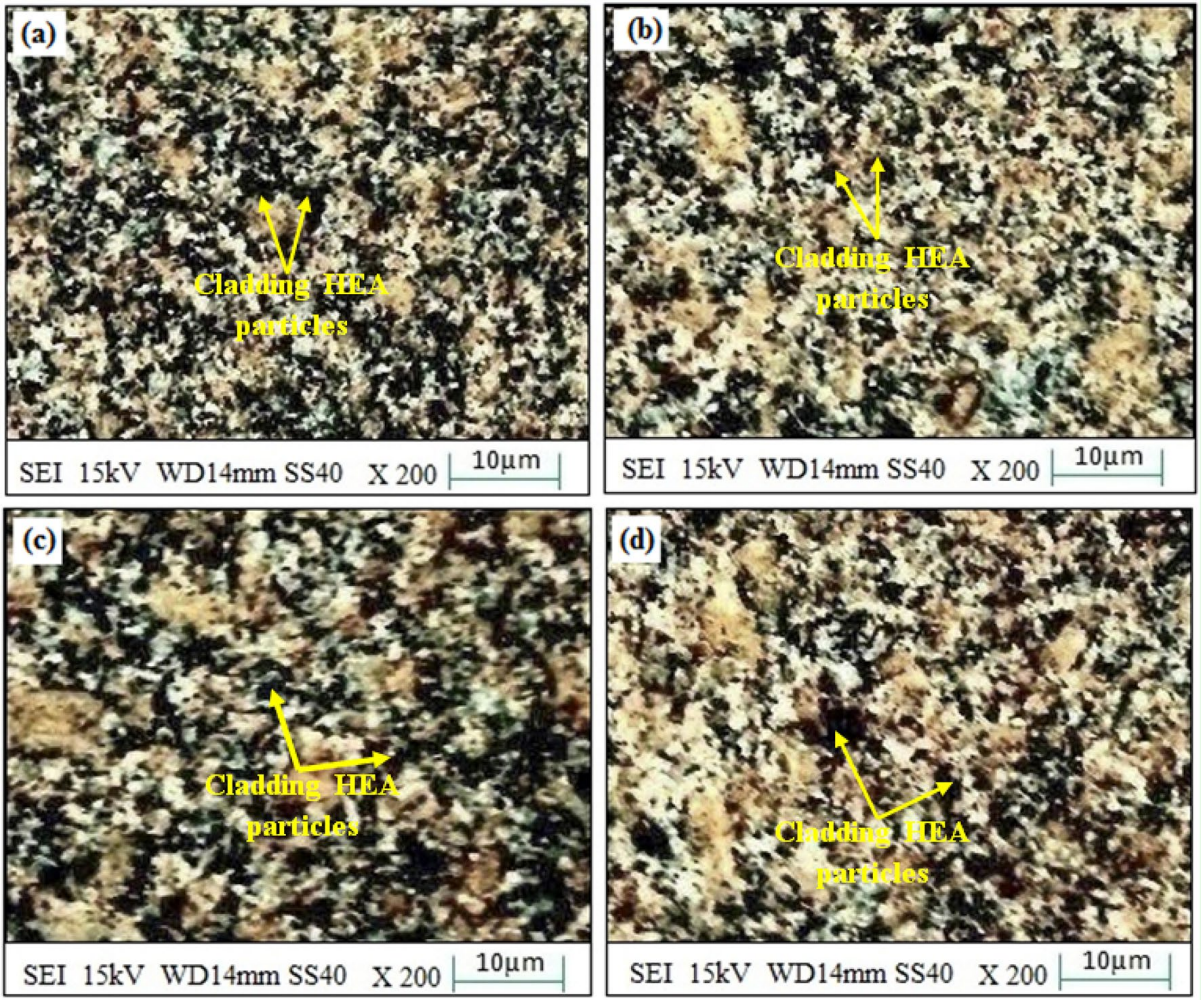
SEM image of SS-304 coating with Fe20Co20Ni20Mn20Cu20 HEA after thermal exposure in the air for up to 10 weeks at 800 °C.

All in all, in the majority of “ferritic-microstructures of steels” (without cladding of 20% Fe 20% Co 20% Ni 20% Mn 20% Cu), transition to “pearlite”, and “ferrite” occurs. In this work, after the “microwave cladding” of 20% Fe 20% Co 20% Ni 20% Mn 20% Cu, there is a considerable deal of uncertainty regarding the “grain-size of the ferrites” owing to the “spacing between interlamellar”, and the “pearlite” does not significantly lend itself to the “strength of cladding-compositions”. In the course of the solidification following “microwave processing”, the “ferrite grains” have been identified in samples of cladding. The size of the ferrite's grain has a considerable impact on the physicomechanical characteristics. Finer the ferrite grain sizes have typically resulted in enhanced mechanical performance^25–28^. There are influencing factors from exterior-sources on both (the "retained-strain" and the final "austenite grain-size") from the "cooling-rate", "alloy-composition", and "deformation-history". Although, the finer grain sizes are typically supported by faster cooling-rates, however the alloy's composition and deformation history also perform crucial functions^43,44^. As reported from the Fig. 12, it has been unveiled that the “size of the ferrite-grains of steel” subsequent to coating has been altered after thermal exposure to the cladding samples of SS-304 coating with 20% Fe 20% Co 20% Ni 20% Mn 20% Cu in the air for up to 10 weeks at 800 °C as shown in Fig. 12. After thermal exposure of Fe20Co20Ni20Mn20Cu20 HEA coating steel, the protective layer was formed in the form of Cr_2_O_3_ and (Fe,Co,Ni,Mn,Cu)_3_O_4_, due to which its corrosion resistance and wear resistance properties enhanced. The alloy's elements (Fe, Co, Ni, Mn, and Cu) can react with atmospheric oxygen during the steel's thermal-exposure to the Fe20Co20Ni20Mn20Cu20 HEA coatings. As a result, oxide-layers initiate to form on the coating's surface. The composition of the alloy has thereby affected the type of oxide formation that results in Cr_2_O_3_ and (Fe, Co, Ni, Mn, Cu)_3_O_4_. A protective-layer of chromium oxide (Cr_2_O_3_) is further potential to form when chromium (Cr) is present^25–28^. Additionally, another alloying element including, (Fe, Co, Ni, Mn, Cu)_3_O_4_ can aid in the formation of spinel oxides. During the mechanism of the protective layer, the Cr_2_O_3_ layer has served as a barrier to prevent further oxidation and corrosion of the underlying material. This layer is illustrious for its capacity to regenerate new protective oxide layers in response to any damage to the existing oxide layer^25–28^. In addition, the coated steel's corrosion resistance is enhanced by the development of a protective oxide layer. This layer has served as a barrier to cease corrosive substances like moisture and aggressive ions from penetrating the substrate below. Moreover, the protective oxide layer's presence can also lead to enhanced resistance to wear. The oxide layer can improve durability and lower material loss by reducing wear and friction between surfaces. A favorable correlation was revealed between the current investigation's findings with Qingqing Zhao et al.^1^.

Furthermore, the SEM images as reported from the Fig. 11 have depicted a consistent homogenous evenly-dispersed cladding layer characterised by a reduced number of dark-pixels, which eventually signifies the enhanced uniformity. The microstructural changes occurred from the higher-temperature austenite-phase to the lower-temperature phase as an outcome of controllable microwave process. This study has contemplated on the ferrite particle-size subsequent to microwave cladding which certainly is one of the crucial contributions towards the strength of the material. As a consequence of microwave cladding, distinctive ferrite-grains developed, which profoundly influenced the material's physicomechanical characteristics favorably^45,46^.

As unveiled from the Fig. 12, a protective shielding chromium-oxide layer as well as a high-entropy spinel-coating composed of (Fe, Co, Ni, Mn, Cu)_3_O_4_ have been developed subsequent to thermal-exposure at 800 °C. The thermal-exposure caused the ferrite grain-size of the steel coating, has revealed that interlamellar-spacing as well as pearlite-formation has made contributions towards this transformation^47–49^. These protective-shielding layers or barrier-coatings are crucial for enhancing the characteristics of resistance to corrosion as well as resistance to wear^50–52^.

**Figure 13:**
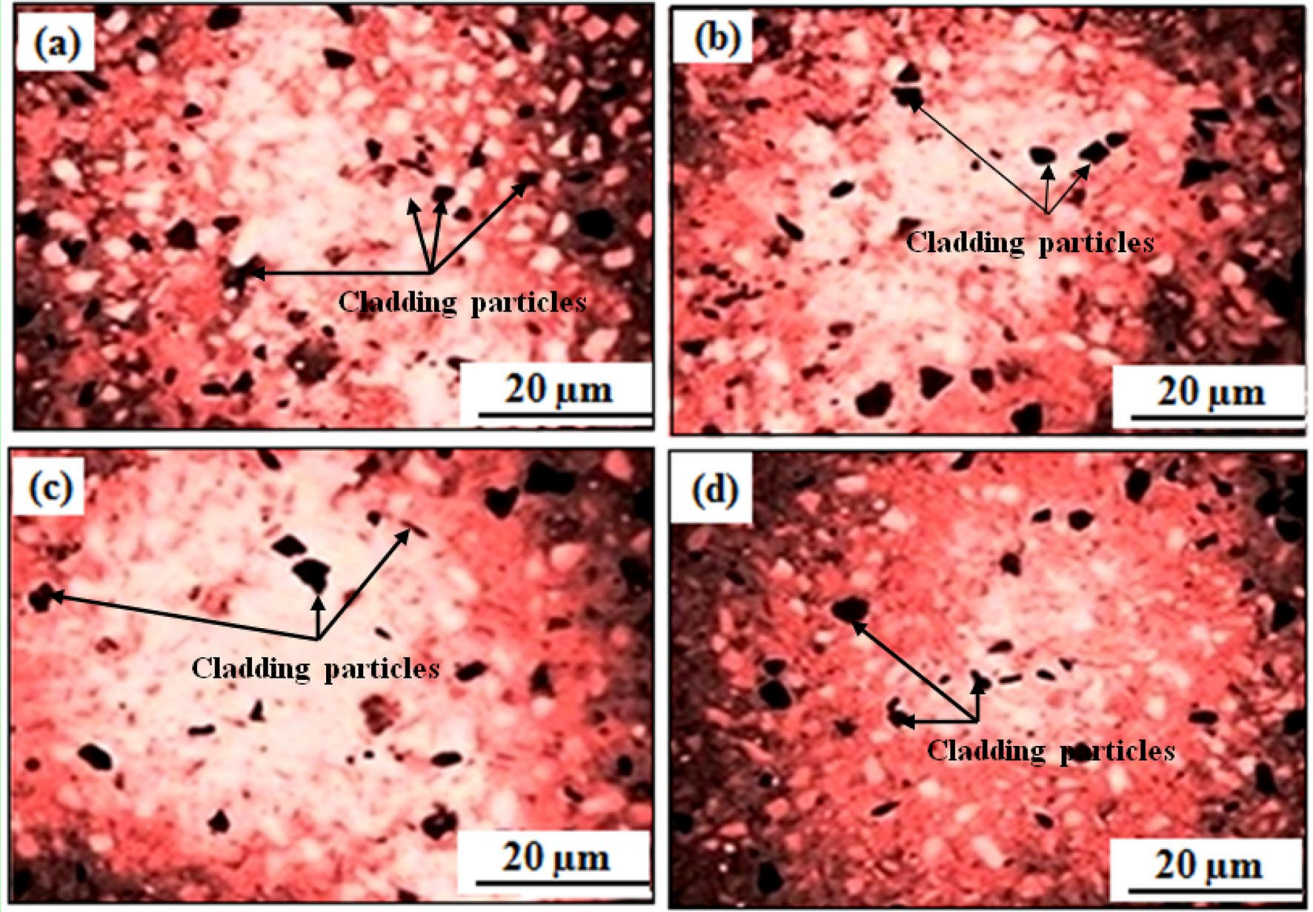
Optical microscopic images of the cladding surface of SS-304 coating with Fe20Co20Ni20Mn20Cu20 HEA, (**a,b**). Before thermal exposure in the air for up to 10 weeks at 800 °C; and (**c,d**). After thermal exposure in the air for up to 10 weeks at 800 °C.

Figure 13a–d has unveiled the optical microscopic view of the cladding samples of SS-304 coating with 20% Fe 20% Co 20% Ni 20% Mn 20% Cu before and after thermal exposure in the air for up to 10 weeks at 800 °C. The Fe20Co20Ni20Mn20Cu20 HEA is coated onto the SS-304 substrate using a microwave cladding process, which initially interacts by melting and fusing the alloy to the substrate. At 800 °C, alloying elements from the cladding composition can diffuse into the substrate, and the substrate's constituents can diffuse into the cladding. Solid solutions or intermetallic phases may form at the interface as a result of this diffusion. Diffusion, phase-transitions, and interactions between the alloy and substrate cause the morphology of the coated sample to change as it is heated to 800 °C^52–54^. The uniformly-distribution along with proper-wettability of entropy alloys (Fe-Co-Ni-Mn-Cu) with SS-304 can be observed. A uniform and defect-free coating has been attained that has revealed the proper wettability, well bonded cladding cladding-layer, superior interfacial-adhesion, robust bonding-strength, and good-contact between the alloy and the substrate^25–28^. In addition, the interaction or interface-adhesion bonding, and dispersion of the alloy elements (Fe, Co, Ni, Mn, Cu) with the SS-304 substrate can be examined through the optical microscopic images. The findings have exhibited that the coatings employed onto the SS-304 substrate has depicted the homogeneity^55–57^. Although, the Fig. 13c,d has been revealed the degree to which the cladding surface which is comprised of the Fe20Co20Ni20Mn20Cu20 HEA composition is capable of withstanding 10 weeks of thermal exposure at 800 °C. Figure 13c,d may reveal that the noticeable shielding layers or protective barriers or protective layers or barriers to damage that develop as a consequence of thermal exposure, including chromium-oxide, and (Fe, Co, Ni, Mn, Cu)_3_O_4_^57–59^. The inclusion of these shielding layers serves as essential role in strengthening resistance to corrosion.

**Figure 14:**
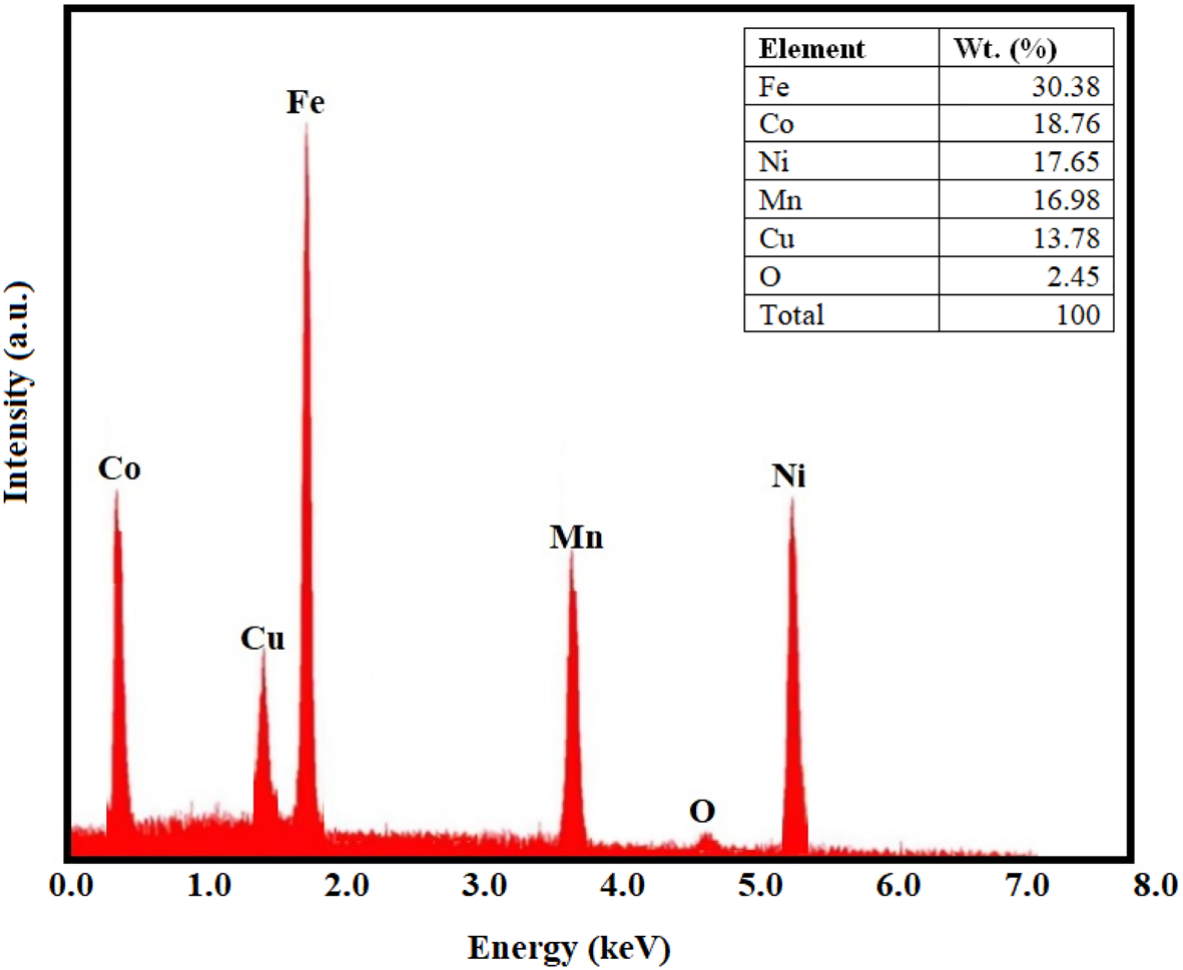
EDS mapping results at the interface region of cladding after thermal-exposure.

Moreover, the EDS outcomes as reported from the Fig. 14 have been employed to discern the particular phases that are generated at the interface. The cladding utilised in this investigation is composed of a blend combination of 20% Fe 20% Co 20% Ni 20% Mn 20% Cu particulates. The EDS findings have provided confirmation that these elements (Fe, Co, Ni, Mn, Cu) have been presented in the cladding layer. The analysis can ascertain whether the 20% Fe 20% Co 20% Ni 20% Mn 20% Cu particulates are dispersed evenly throughout the cladding layer. The study has specified (Fe,Co,Ni,Mn,Cu)_3_O_4_, Cr_2_O_3_, Fe, FeNi_3_, NiCO, Fe_2_O_3_, CuO, and Mn_2_O_3_ phases after thermal-exposure. This substantiates the assertion regarding the development of resilient, hard, stable, durable, high strength, tough, and robust protective barrier layer coatings^28,53^.

EDS analysis have affirmed the existence of pertinent elements including Fe, Co, Ni, Mn, Cu and others within the cladding layer. A uniformly-dispersion, and homogeneity arrangement of Ni, TiC, and TiB_2_ elements indicates the presence of a cladding layer that is well bonded, homogenous, and uniform in consistency^28,53^.

The SS-304 substrate as well as the cladding layer's metallurgical bonding can be assessed by examining the EDS findings. Good bonding would be indicated by the absence of different interfaces and void-gaps in elemental dispersion. Metallurgical bonding may have been confirmed through the identification of elements prevalent throughout both the cladding layer as well as the substrate at the interface region^28,53^.

### XRD testing analysis

**Figure 15:**
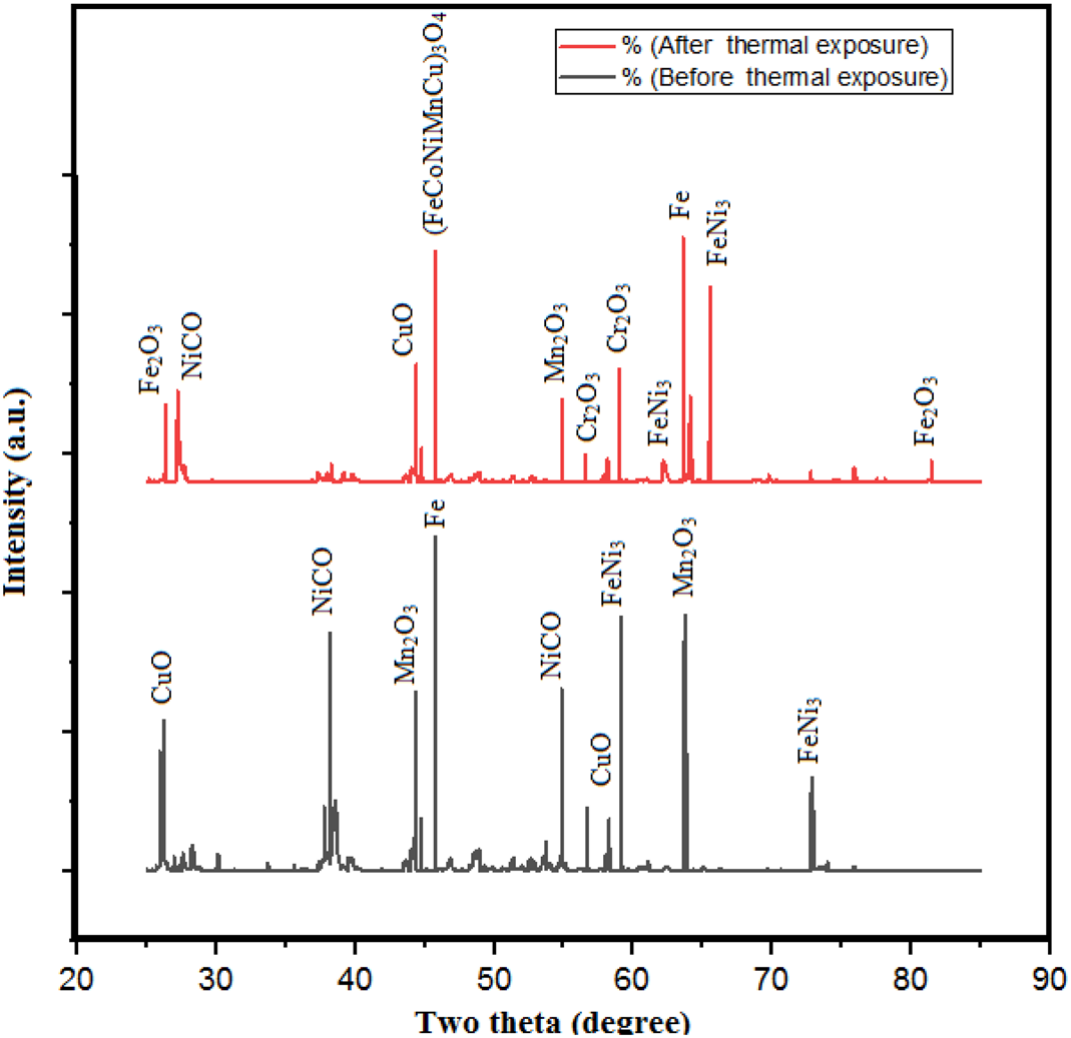
XRD analysis of the clad samples with coating 20% Fe 20% Co 20% Ni 20% Mn 20% Cu on SS-304 before and after the thermal exposure in the air for up to 10 weeks at 800 °C.

Figure 15 has depicted the XRD testing of the clad samples with coating 20% Fe 20% Co 20% Ni 20% Mn 20% Cu on SS-304 before and after the thermal exposure in the air for up to 10 weeks at 800 °C. XRD examination of the cladded surface before thermal exposure has reported the presence of Fe, FeNi_3_, Al_2_O_3_, NiCO, CuO, and Mn_2_O_3_ phases. The Fe phase has contributed to strengthening the strength, durability, stability and resilience of the cladding material. Fe phase has contributed to the mechanical and structural characteristics of the Fe20Co20Ni20Mn20Cu20 HEA by forming the base element. FeNi_3_ phase has served as an indicator of nickel content within the alloy. As indicated by this phase, an intermetallic compound possibly been formed. Nickel has served to enhance the alloy's resistance to corrosion and mechanical strength. Al_2_O_3_ phase has contributed to the formation of the protective layer and is probably generated during thermal-exposure. In addition, Al_2_O_3_ phase has inhibited an additional oxidation by serving as a protective barrier under severe environmental circumstances, an inhibitory layer, a barrier or shielding layer to prevent damage. The stability and hardness at elevated temperatures are characteristics offered by this phase. The NiCO phase has characterised the material's composition by indicating the existence of nickel as well as carbon. This phase has potentially aided in corrosion resistance. Copper content is indicated by this CuO phase. Copper potentially has enhanced both resistance to corrosion as well as electrical conductivity. Mn_2_O_3_ phase has provided an indication of manganese content within the alloy. The strength, hardness, resilience, and durability of the alloy has been enhanced by the presence of manganese.

While, XRD examination of the cladded surface after the thermal exposure has revealed the presence of (Fe,Co,Ni,Mn,Cu)_3_O_4_, Cr_2_O_3_, Fe, FeNi_3_, NiCO, Fe_2_O_3_, CuO, and Mn_2_O_3_. Additionally, the JCPDS number 65-3178 uniquely identified the crystal structure in X-ray powder diffraction analysis for precise material characterization. The (Fe,Co,Ni,Mn,Cu)_3_O_4_ phase has served as a high-entropy spinel-coating for SS-304^57–59^. This phase has exhibited resistance to oxidation and corrosion by acts as an inhibitory shield for SOFC interconnects. Cr_2_O_3_ phase has performed the function of establishing a barrier-layer or shielding coating on the surface. Anticorrosion and abrasion resistance are the enhanced characteristics that have been contributed by Cr_2_O_3_ phase. Although, the Fe, FeNi_3_, NiCO, CuO, and Mn_2_O_3_ phases are potentially aiding in the enhancement of hardness, strength, and durability. The hardening of the material is facilitated by the formation of these phases subsequent to microwave cladding^57–59^. Fe shows the presence of crystal phase of Iron, while, the Fe_2_O_3_ shows the formation due to oxidation. Resistance to oxidation and corrosion of the coated SS-304 has been influenced by the Fe_2_O_3_ phase. The structural integrity, stability, and strength of the cladding layer could potentially be enhanced by the inclusion of Fe_2_O_3_ in the coating. Fe_2_O_3_ phase is appropriate for applications involving thermal exposure due to its resistance to withstand at an elevated temperature. According to the research's emphasis on thermal exposure at 800 °C, the stability of the coating at elevated temperatures could potentially be attributed to the existence of the Fe_2_O_3_ phase. The presence of protective-layers, which enhances the durability of the material, can be facilitated by iron(III) oxide or hematite. By forming a barrier against additional oxidation, Fe_2_O_3_ has potentially served as a corrosion-resistant layer. In high-temperature as well as corrosive-conditions, Fe_2_O_3_ phase has the potential to enhanced the overall protective characteristics of the coatings. Enhancing the overall effectiveness, performance-efficacy, and functional-efficiency of the coating is potentially achievable as an outcome of the chemical long-term stability, chemically-resistance, as well as hardness of Fe_2_O_3_ phase. Hence, the findings revealed that the formation of (Fe,Co,Ni,Mn,Cu)_3_O_4_ phase is potential coating material for SOFC interconnects^57–59^.

### Surface hardness

**Figure 16:**
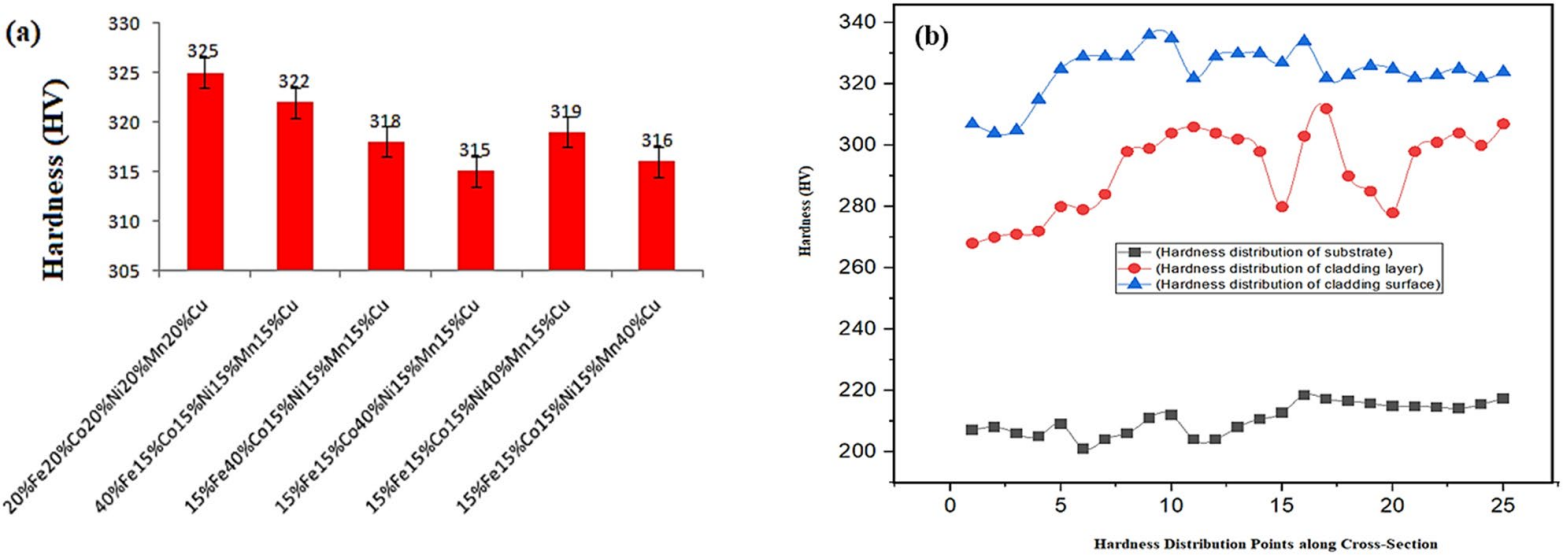
(**a**) Surface hardness of the SS-304 alloy cladding with distinct formulated samples, and (**b**). Hardness Distribution along Cross-Section of Substrate, Cladding layer, Cladding Surface.

The “hardness” of the material is known as the main property of any surface of the material. So as per the investigation, the “average hardness” of SS-304 is 210 HV, and the “hardness” of the cladding-surface of SS304 with the mixture of 20% Fe 20% Co 20% Ni 20% Mn 20% Cu was about 325 HV as portrayed in Fig. 16a. The results have unveiled about 54.76% improvement in the “hardness of SS-304 alloy” after the “microwave cladding” with a mixture of 20% Fe 20% Co 20% Ni 20% Mn 20% Cu. thus, the “microwave-cladding” has resulted in the production of numerous harder-phases, including, FeNi_3_, Al_2_O_3_, NiCO, CuO, and Mn_2_O_3_ phases may be responsible for strengthening the hardness of steel^57–59^.

The hardness-distribution profile variation patterns or curves for SS-304, the cladding-layer, and the cladding-surface were analysed and are depicted in Fig. 16b. It is notable that the degree of hardness pattern distributions for the cladding-layer as well as cladding-surface were homogenous uniformity, indicating that the material characteristics maintained consistent. Contrary to this, SS-304 substrate has demonstrated the substantial variation in hardness subsequent to solidification. It is noteworthy that the hardness for the cladding layer was slightly lower in comparison to that of the cladding surface^57–59^. The discernible characteristics conferred upon every region seemed the consequence of the microwave cladding method, as evidenced by the differences in the degree of hardness profile.

### Corrosion behavior

**Figure 17:**
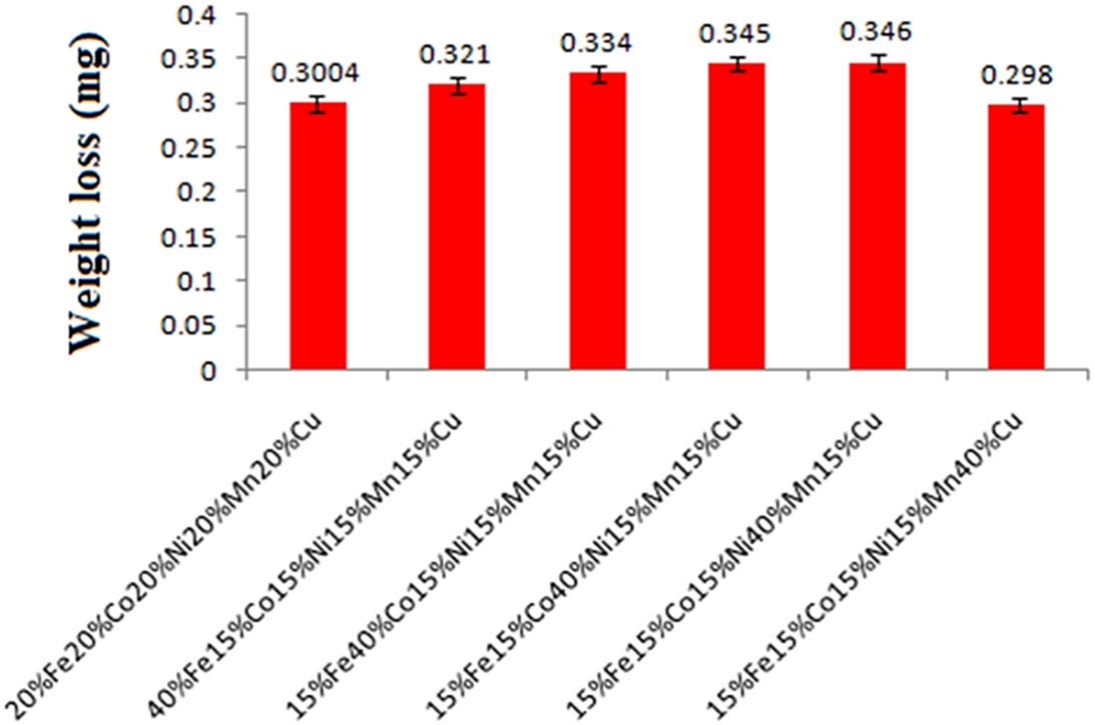
Weight loss after corrosion test of the SS-304 alloy cladding with distinct formulated samples.

Corrosion test of SS-304 coating with 20% Fe 20% Co 20% Ni 20% Mn 20% Cu before and after thermal exposure in the air for up to 10 weeks at 800 °C has been conducted for 120 h in 3.5 wt% NaCl media. As the corrosion weight loss is the total amount of material lost as an outcome of corrosion over a predetermined specific-period as reported from the Fig. 17. The weight loss of the sample composition for the blend-combination with 20% Fe 20% Co 20% Ni 20% Mn 20% Cu coatings was discovered to be 0.3004 mg as depicted in Fig. 17. Nevertheless, the weight loss for 15% Fe 15% Co 15% Ni 15% Mn 40% Cu coating sample was found to be 0.298 mg as reported from Fig. 17. Although, the development of an oxide surface raises the probability that the surface has reacted with the environment, which has raised the possibility that corrosion has developed^57–59^. The influence of two key parameters is responsible for the corrosion µresistance and self-effacing resistance of coatings. Moreover, the (Fe,Co,Ni,Mn,Cu)_3_O_4_ phase has served as a high-entropy spinel-coating for SS-304^57–59^. This phase has exhibited resistance to oxidation and corrosion by acts as an inhibitory shield for SOFC interconnects. Cr_2_O_3_ phase has performed the function of establishing a barrier-layer or shielding coating on the surface. Anticorrosion and abrasion resistance are the enhanced characteristics that have been contributed by Cr_2_O_3_ phase. Although, the Fe, FeNi_3_, NiCO, CuO, and Mn_2_O_3_ phases are potentially aiding in the enhancement of hardness, strength, and durability. Using the microwave cladding energy technique, a corrosion-resistant layer has been developed that is well bonded. The cladded material's weight loss due to corrosion is reduced as a result, making it suitable for use in corrosive environments where protection from chemical deterioration is crucial. These coatings are also very resistant to high temperatures, which makes them consummate for SOFC interconnect applications. Steel's hardness can be significantly increased with the aid of FeNi_3_, Al_2_O_3_, NiCO, CuO, and Mn_2_O_3_ phases in coatings, which has a positive impact on performance in high-wear applications.

**Figure 18:**
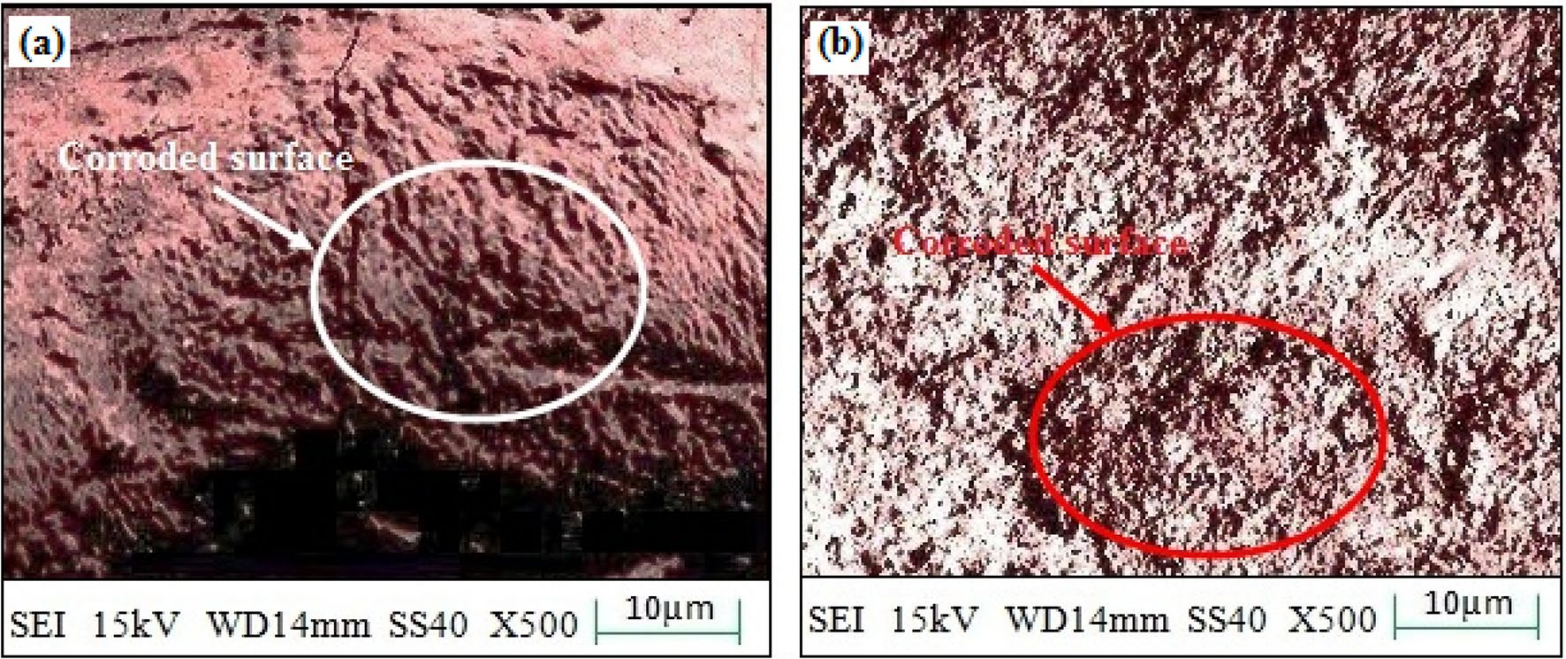
(**a**,**b**): Surface morphology of corroded SS-304 without coating the mixture of 20% Fe 20% Co 20% Ni 20% Mn 20% Cu.

Additionally, Fig. 18a,b has reported the surface morphology of corroded SS-304 without coating the mixture of 20% Fe 20% Co 20% Ni 20% Mn 20% Cu. Significant corrosion behavior in SS-304 steel can be observed without coating 20% Fe 20% Co 20% Ni 20% Mn 20% Cu. The passive oxide-layer on the surface of SS-304 may become compromised at high temperatures and in the presence of chloride-ions. This may result in homogenously corrosion, in which the surface corrodes uniformly throughout. When corrosion products like iron oxides and chlorides form, the surface may become rough and lose its original glistening surface-texture. The localized variations in the protective oxide layer may, in certain circumstances, cause pitting corrosion to commence^57–59^. Pits are tiny cavities that can delve deeply into the substance. Chloride ions can stimulate the oxidizing layer's breakdown, which could result in a localized-attack^57–59^. Pitting corrosion can cause localized, irregular, uneven, and erratic surface roughness. The SS-304 steel may experience oxidation reactions at high-temperatures, resulting in the development of iron-oxide scales on the surface. This may also increase surface-abrasion and alter morphology.

**Figure 19:**
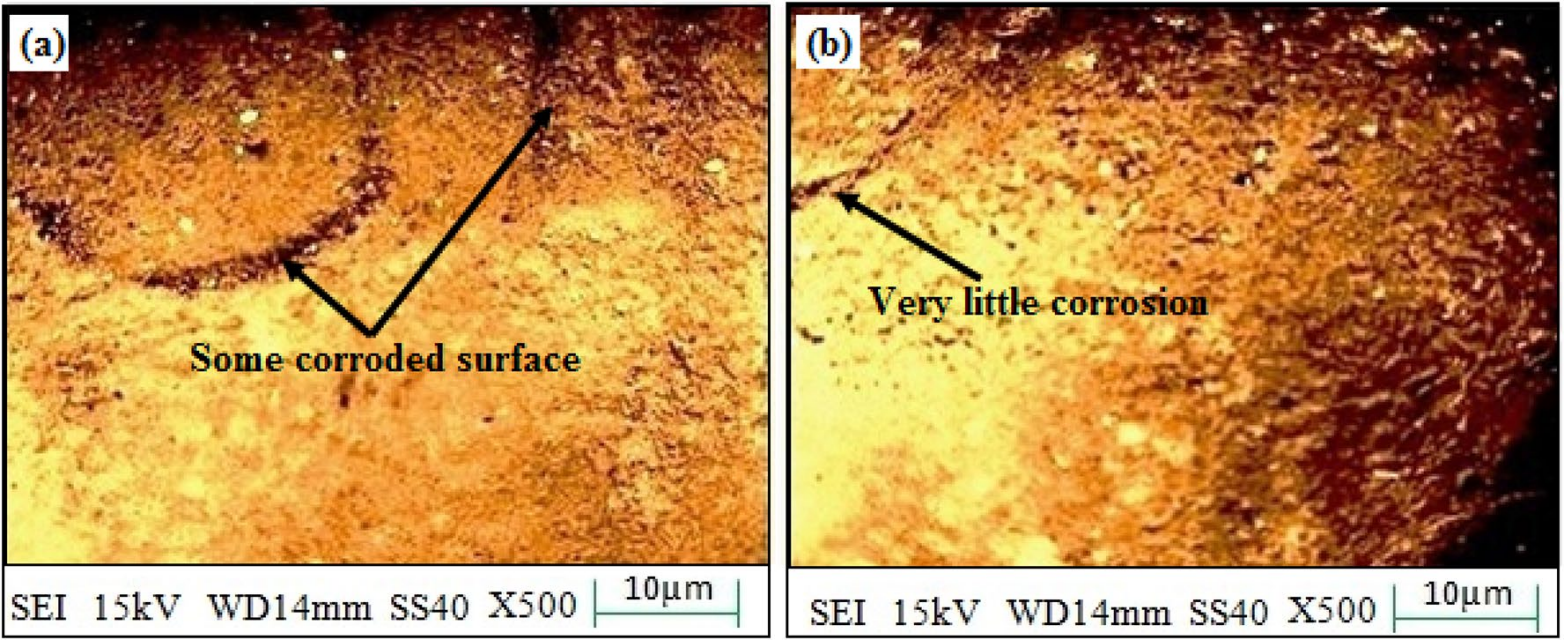
(**a**,**b**): Surface morphology of corroded SS-304 with coating the mixture of 20% Fe 20% Co 20% Ni 20% Mn 20% Cu; (**a**) before the thermal exposure in the air for up to 10 weeks at 800 °C, (**b**) after the thermal exposure in the air for up to 10 weeks at 800 °C.

The corroded surface of SS-304 may exhibit some dark spots with coating the mixture of 20% Fe 20% Co 20% Ni 20% Mn 20% Cu as shown in Fig. 19). However, corrosion loss was reduced after the thermal exposure in the air for up to 10 weeks at 800 °C as shown in Fig. 19b. The Fe, Co, Ni, Mn, and Cu are combined to produce the coating, which is employed as a protective layer on the surface of SS-304 steel. These alloying components can enhance corrosion-resistance and serve as sacrificial anodes to shield the underlying SS-304 steel by being present in the coating. When subjected to corrosive conditions (3.5 wt. % NaCl media) the initial corrosion processes commence. The development of dark-spots on the surface of SS-304 is conceivably caused by chloride-ions that can penetrate the coating and commence localized corrosion reactions^57–59^. These dark areas could be indications of localized corrosion, such as pitting or crevice corrosion, where the protective oxide layer on the SS-304 may have been compromised or where the coating may have been breached. The corrosion processes can be considerably accelerated by the high temperature exposure (800 °C). It may encourage the diffusion of corrosive species into the SS-304 substrate and through the coating. Increased corrosion rates and improved chemical reactions may result from this. Different rates of corrosion may be the cause of the dark spots that were observed on the SS-304 surface that had been coated. There may be alterations in the rate of corrosion across the surface as a result of the coating's inequitable protection. Dark areas could be regions where the coating has performed less well at preventing corrosion, possibly as a result of flaws or microstructural alterations^57–59^. Unexpectedly, the corrosion loss is decreased after a 10-weeks thermal exposure at 800 °C. This decrease may be caused by various factors:

The development of protective oxide scales on the surface of SS-304 may be aided by the high temperature. These scales can function as barriers, retardation the diffusion of corrosive species and offering a modicum of protection against additional corrosion. The alloying elements (Fe, Co, Ni, Mn, and Cu) could help form stable oxide scales in the coating, which has contributed to mitigate the corrosion susceptibility. In addition, the continuous exposure to high temperatures and corrosive environments may result in the development of a stronger passive oxide layer on the surface of SS-304, furthermore preventing corrosion^57–59^. Even if initially compromised, the coating may moderately obstruct the diffusion of corrosive ions, deceleration the rate of corrosion over time. Although, the corrosion typically progresses to an equilibrium state over time, where the initial quick corrosion may retard down as the system acclimates to the corrosive circumstances^57–59^.

### Wear behavior

**Figure 20:**
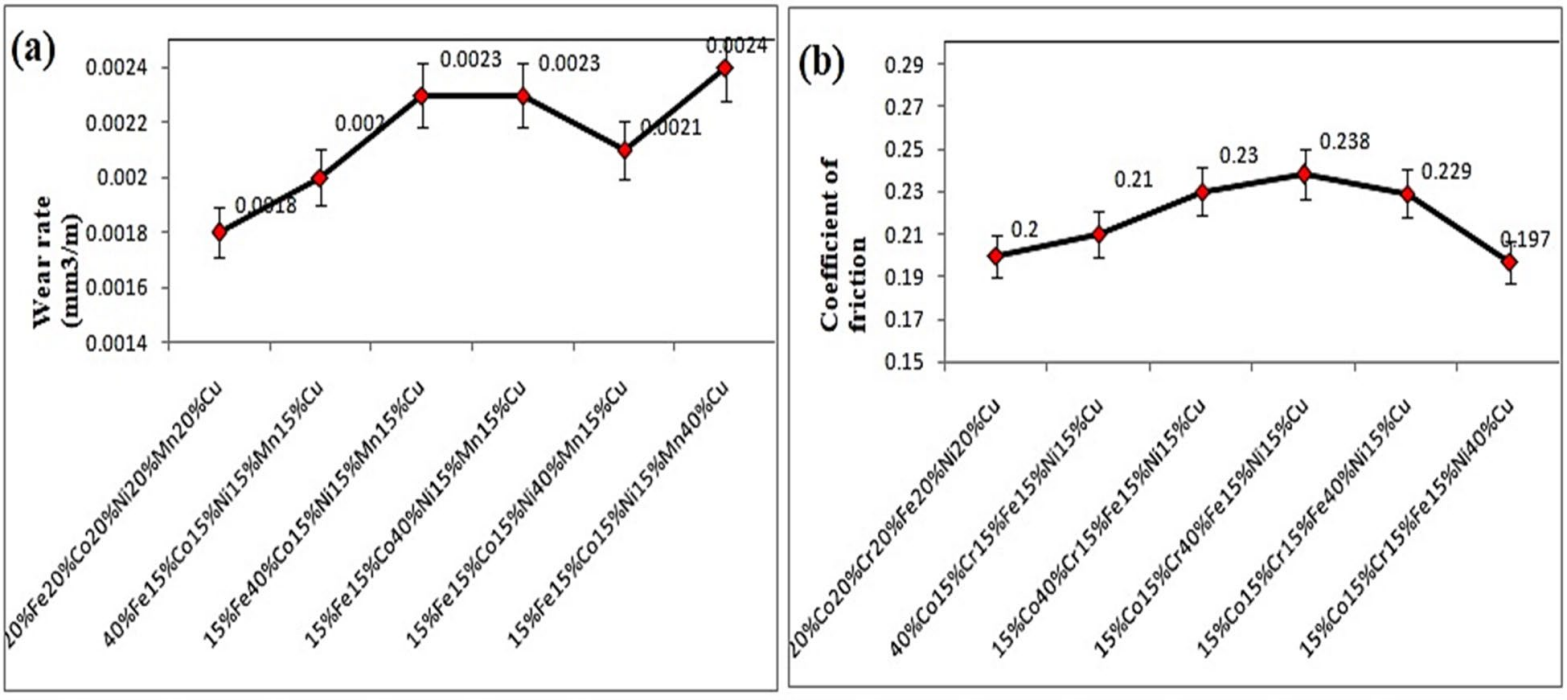
(**a**) Wear rate, (**b**) Coefficient of friction of the SS-304 alloy cladding with distinct formulated samples.

The wear behavior of microwave-clad samples was scrutinized under specific conditions: a sliding speed of 2 m/s, a sliding distance of 1000 m, and a load of 15 N. Notably, a coating comprising 20% Fe, 20% Co, 20% Ni, 20% Mn, and 20% Cu applied to steel exhibited a minimal wear rate, measuring at 0.0018 mm^3^/m as illustrated in the Fig. 20a. This observation suggests the effectiveness of the coating in enhancing the wear resistance of the steel substrate^57–59^. The coefficient of friction for a steel surface coated with a cladded-particulates comprising 20% Fe, 20% Co, 20% Ni, 20% Mn, and 20% Cu was determined to be 0.2 as depicted in the Fig. 20b. This finding has indicated a moderate frictional resistance, suggesting potential applications where balanced friction properties are desired for optimal performance.

**Figure 21:**
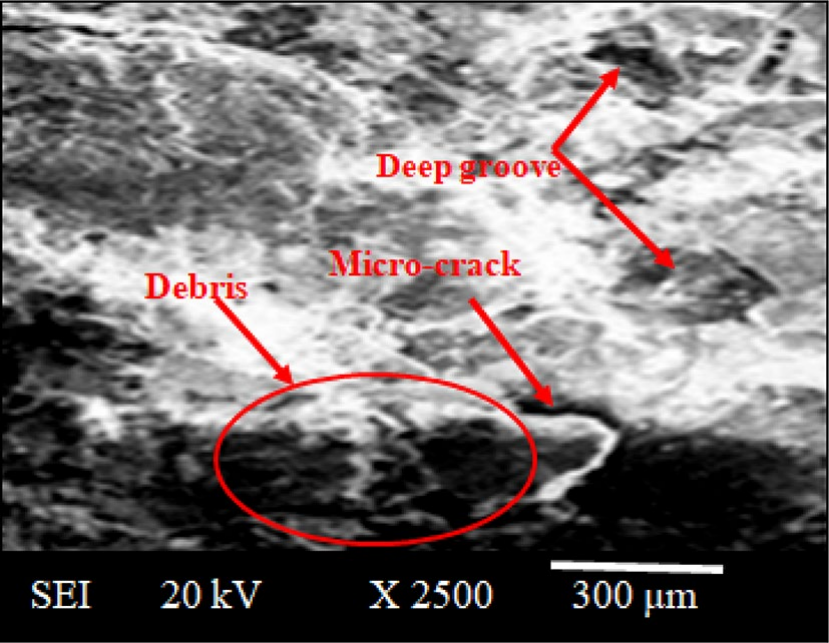
SEM image of worn-surface of SS-304 cladding with 20% Fe, 20% Co, 20% Ni, 20% Mn, and 20% Cu.

In Fig. 21, the SEM images have depicted the worn surface of SS-304 cladding with a composition of 20% Fe, 20% Co, 20% Ni, 20% Mn, and 20% Cu. Notably, the image reveals the presence of wear debris, deep grooves, and micro-cracks on the surface. These features suggest the complex nature of the wear mechanism and highlight the interactions between the cladding material and the external-forces. The SEM analysis provides valuable insights into the wear characteristics, aiding in a comprehensive understanding of the tribological performance of the microwave cladded coatings, essential for optimizing materials in various engineering applications^57–59^.

### Thermal expansion test for thermal stability of cladding coatings for SOFC interconnects applications:

**Figure 22:**
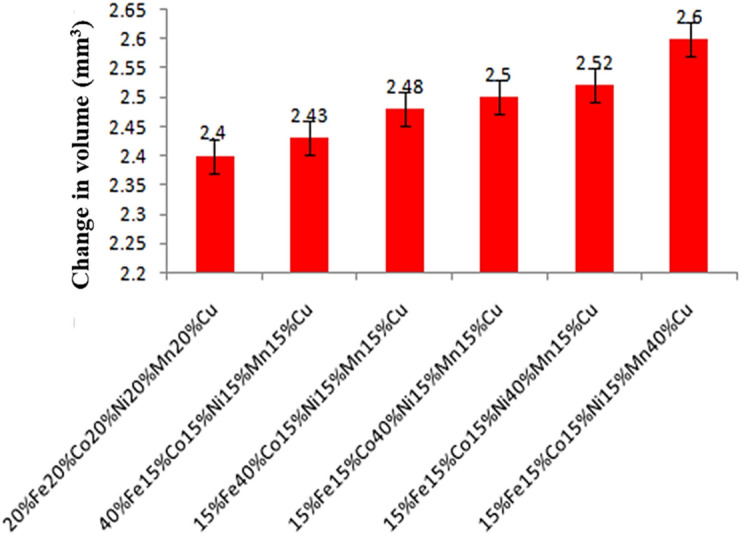
Change in volume after thermal expansion test of the SS-304 alloy cladding with distinct formulated samples.

The thermal stability of cladding samples played a crucial role in assessing material integrity under elevated temperatures for SOFC-interconnect applications. A thorough examination involved subjecting the samples to a thermal expansion test in a muffle-furnace, maintaining a temperature of 1200 °C for a 72 h duration. Notably, the cladding of stainless steel (SS304) with a composition of 20% Fe, 20% Co, 20% Ni, 20% Mn, and 20% Cu demonstrated remarkable stability. The results revealed a minimal change in volume, with only a 1 mm^3^ variation observed as exhibited in the Fig. 22. This suggests that the composite cladding effectively resisted significant alterations in volume during prolonged exposure to high temperatures, showcasing its robust thermal stability^57–59^. Such findings are pivotal in SOFC-interconnect applications where materials must withstand elevated temperatures without compromising structural-integrity, highlighting the potential suitability of the analysed composite for demanding thermal environments^57–59^. The study has contributed valuable insights into the thermal behavior of materials, essential for engineering applications where stability under extreme temperatures is a critical factor.

In comparison with prior literary-studies, the practical implications for this study have been elucidated as follows, following the microwave cladding with Fe20Co20Ni20Mn20Cu20 HEA, the hardness of SS-304 has been raised around 54.76%^57–59^. The real-world implications of this have significant impacts in sectors that depend primarily on hardness and resistance to wear and corrosion. Subsequent to thermal treatment at 800 °C, the material forms a protective chromium oxide layer as well as a high-entropy spinel cladding that eventually contributed for applications in severe environments, such as SOFC interconnects. The microwave-cladded coatings that have been developed has furthermore exhibited prospective potential components in SOFC interconnects. By strengthening mechanical characteristics, stability at elevated temperatures, and resistance to corrosion, the Fe20Co20Ni20Mn20Cu20 HEA composition as well as microwave cladding method has effectively accomplished the crucial requirements for SOFC interconnect materials. The research has underlined the precision and controlled microwave-assisted alloying of Fe20Co20Ni20Mn20Cu20 HEA onto SS-304 steel. Hence, the material and energy consumption benefits associated with this controlled technique rendering it a viable and effective technique for manufacturing^57–59^.

Additionally, an interconnect for SOFCs has served to establish mechanical and electrical connection-links among individual cells comprising a fuel-cell array. The efficacy, durability, stability, performance-functioning, and long-term viability of the SOFC system are profoundly affected by the interconnects. Several criteria must be fulfilled with an array of prerequisites as elucidated follows. In an attempt to facilitate the movement passage of electrons produced during the electrochemical reactions that occur within the fuel cell, SOFC interconnects must possess an elevated electrical conductivity. Thus, effective power-generation is ensured. The thermal expansion coefficient for the materials employed for interconnects in SOFCs must be in close proximity or vicinity with that of the adjacent components of the cell, considering the extremely higher-temperatures in which these cells function in practice^57–59^. Mechanical stress and fractures are thereby mitigated during the process of thermal-cycling. Throughout the functioning of SOFCs, interconnects undergo exposure to corrosive and oxidative atmosphere circumstances. Maintaining the integrity of their structure as well as ability to conduct electricity for a prolonged period necessitates resistance to corrosion and oxidation^57–59^. Typically, distinct gaseous concentrations have been employed on each side of the interconnect in SOFCs, which impacts gas tightness. Gas tightness is a vital characteristic for the interconnect material as it serves to act as a barrier to or to inhibit cross-contamination as well as promise the fuel cell's optimal operation, maximal efficiency, and optimum performance functioning.

For the purpose of determining whether SS-304 steel can be utilised as a SOFC interconnect material, the deposition of a Fe20Co20Ni20Mn20Cu20 HEA utilising microwave energy is investigated in the context of your research. Prior to thermal exposure at elevated temperatures, the alloy is deposited onto SS-304, a stainless steel renowned for its resistance to corrosion.

## Conclusions

The present study yields the following conclusions:

i. The blend combination of Fe20Co20Ni20Mn20Cu20 HEA powder of 20% Fe 20% Co 20% Ni 20% Mn 20% Cu can be utilized on “SS-304 alloy” for “microwave cladding” via., “microwave energy”.
ii. SEM image of SS-304 alloy with the “microwave cladding” of the mixture of Fe20Co20Ni20Mn20Cu20 HEA powder of 20% Fe 20% Co 20% Ni 20% Mn 20% Cu has showed uniform cladding of the particles along the surface.
iii. The uniformly “cladding surface”, and “cladding layer” were attained by retaining the optimal microwave factors. An enhanced degree of homogeneity/uniformity in the "cladding surface", and "cladding layer" has reduced the "deviation from geometric dimensions" of the “cladding surface”.
iv. After the thermal-exposure of deposited steel with 20% Fe 20% Co 20% Ni 20% Mn 20% Cu in the air for up to 10 weeks at 800 °C, a “protective Cr_2_O_3_ layer”, and “high-entropy spinel coating of (Fe,Co,Ni,Mn,Cu)_3_O_4_” have been formed.
v. The hardness has been ameliorated by about 54.76%. The foremost cause of the enhancement in hardness is the apparent observable distinct harder-phases formed after the “microwave-cladding”, including Fe, FeNi_3_, Al_2_O_3_, NiCO, CuO, Fe_2_O_3_, and Mn_2_O_3_ phases. Although, the Fe has depicted the presence of crystal phase of Iron, while, the Fe_2_O_3_ has exhibited the formation due to oxidation.
vi. The “corroded-surface” of SS-304 with coating the mixture of Fe20Co20Ni20Mn20Cu20 HEA powder 20% Fe 20% Co 20% Ni 20% Mn 20% Cu has exhibited few dark spots. However, corrosion loss was reduced after thermal exposure in the air for up to 10 weeks at 800 °C.
vii. As evidence of its robust thermal stability for SOFC-interconnect applications, the outcomes of the study illustrate that the composite cladding satisfactorily withstand considerable volumetric changes throughout a prolonged exposure to extremely high temperatures.

## Future outlook

i. Emphasis must be contemplated on analysing the electrical-conductivity for SOFCs interconnects applications.
ii. Emphasis must be centered on analyzing the “oxidation-resistance”, and “area-specific resistance” in the simulated service environments for applications in SOFCs.
iii. Additional characterizations and analysis must be contemplated in an extensive manner on the coatings before and after thermal exposure in air at 800℃.
iv. The cross-section of the coatings has been assessed to affirm the continuous and protective (FeCoNiMnCu)_3_O_4_ coating employing characterization analysis.

## Data Availability

The data that support the findings of this study are available within this manuscript.

## Abbreviations

HEAs: High entropy alloys
SS-304: Stainless Steel 304
SOFC: Solid oxide fuel-cell
SEM: Scanning electron microscope
XRD: X-ray diffraction
MHH: Microwave hybrid heating
BPR: Ball milling speed-to-powder ratio
CCA: Composition-alloy complex
NaCl: Sodium chloride
Fe: Iron
Co: Cobalt
Ni: Nickel
Mn: Manganese
Cu: Copper
Cr_2_O_3_: Chromium oxide
(Fe, Co, Ni, Mn, Cu)_3_O_4_: High-entropy spinel coating
wt%: Weight percentage
MPa: Megapascal
GHz: Gigahertz
HV: Vickers hardness

## References

[1] Zhao Q (2022). High-entropy FeCoNiMnCu alloy coating on ferritic stainless steel for solid oxide fuel cell interconnects. J. Alloys Compd..

[2] Gupta D, Sharma AK, Bhovi PM, Dutta S (2012). Development and characterization of microwave composite cladding. J. Manuf. Process..

[3] Zafar S, Sharma AK (2016). Structure-property correlations in nanostructured WC–12Co microwave clad. Appl. Surf. Sci..

[4] Hebbale AM, Srinath MS (2016). Microstructural investigation of Ni based cladding developed on austenitic SS-304 through microwave irradiation. J. Mater. Res. Technol..

[5] Fernandez JB (2018). Tribological behavior of AA1050H24-graphene nanocomposite obtained by friction stir processing. Metals.

[6] Sharma AK, Aravindhan S, Krishnamurthy R (2011). Microwaveglazing of alumina–titania ceramic composite coatings. Mater. Lett..

[7] Zhou S, Zeng X, Qianwu H, Huang S (2008). Analysis of crack behavior for Ni-based WC composite coatings by laser cladding and crack-free realization. Appl. Surf. Sci..

[8] Leonelli RC, Veronesi P, Denti L, Gatto A, Iuliano L (2008). Microwave assisted sintering of green metal parts. J. Mater. Process. Technol..

[9] Mateos J, Cuetos JM, Fernandez E, Vijande R (2000). Tribological behavior of plasma-sprayed WC coatings with and without laser remelting. Wear.

[10] Roy Agrawal D, Cheng J, Gedevanishvili S (1999). Full sintering of powdered-metal bodies in a microwave field. Nature.

[11] Mondal A, Upadhyaya A, Agrawal D (2009). Microwave sintering of refractory metals/alloys. J. Microw. Power Electromagn. Energy.

[12] Chhillar P, Agrawal D, Adair JH (2008). Sintering of molybdenum metal powder using microwave energy. Powder Metall..

[13] Zambon A, Ramous E (1993). Laser beam energy absorption enhancement by means of coatings. Laser Eng..

[14] Singh B, Zafar S (2021). Microwave cladding for slurry erosion resistance applications: A review. Mater. Today.

[15] Durga Prasad C, Shashank Lingappa M, SharnappaJoladarashi MR, Ramesh BS (2021). Characterization and sliding wear behavior of CoMoCrSi + Flyash composite cladding processed by microwave irradiation. Mater. Today.

[16] Singh B, Zafar S (2020). Understanding time-temperature characteristics in microwave cladding. Manuf. Lett..

[17] Vishwanatha JS, Hebbale AM, Kumar N, Srinath MS, Badiger RI (2021). ANOVA studies and control factors effect analysis of cobalt based microwave clad. Mater. Today Proc..

[18] Phanendra Kumar K, Mohanty A, Shashank Lingappa M, Srinath MS, Panigrahi SK (2020). Enhancement of surface properties of austenitic stainless steel by nickel based alloy cladding developed using microwave energy technique. Mater. Chem. Phys..

[19] Singh B, Zafar S (2021). Influence of post clad heat treatment on microstructure and slurry erosion characteristics of Ni-based microwave clad. Vacuum.

[20] Mishra TK, Kumar A, Sinha SK (2020). Investigation of sliding wear behaviour of Ni-WC microwave cladding. Mater. Today Proc..

[21] Babu A, Arora HS, Singh H, Grewal HS (2019). Microwave synthesized composite claddings with enhanced cavitation erosion resistance. Wear.

[22] Rakesh B, Nair HS, Arora AV, Boyana P, Saiteja HS (2019). Tribological behavior of microwave synthesized high entropy alloy claddings. Wear.

[23] Dwivedi SP, Sharma S, Sharma S (2020). Identification of microwave radiation effect on copper welded joint with brass as filler material using response surface methodology. Mater. Perform. Charact..

[24] Dwivedi SP, Sharma S, Singh T, Kumar N (2020). Mechanical and metallurgical characterization of copper-based welded joint using brass as filler metal developed by microwave technique. Ann. Chim. Sci. Matér..

[25] Dwivedi S, Sharma S, Sharma KP, Kumar A, Agrawal A, Singh R, Eldin SM (2023). The microstructure and properties of Ni-Si-La_2_O_3_ coatings deposited on 304 stainless steel by microwave cladding. Materials.

[26] Singh G (2022). Impact of post-heat-treatment on the surface-roughness, residual stresses, and micromorphology characteristics of plasma-sprayed pure hydroxyapatite and 7%-Aloxite reinforced hydroxyapatite coatings deposited on titanium alloy-based biomedical implants. J. Mater. Res. Technol..

[27] Singh SK (2022). Effect of alumina oxide nano-powder on the wear behaviour of CrN coating against cylinder liner using response surface methodology: processing and characterizations. J. Mater. Res. Technol..

[28] Dwivedi S, Maurya M, Sharma S (2023). Mechanical and microstructure behavior of cladding surface SS 304 coating with Ni and Al2O3 by microwave technique. J. Inst. Eng. India Ser. C..

[29] Dwivedi S, Sharma S (2023). Effect of CeO2–Ni addition on the behavior of AZ91- A356 based composite fabricated by friction solid state technique. Mater. Chem. Phys..

[30] Sharma S, Patyal V, Sudhakara P, Singh J, Petru M, Ilyas RA (2021). Mechanical, morphological, and fracture-deformation behavior of MWCNTs-reinforced (Al–Cu–Mg–T351) alloy cast nanocomposites fabricated by optimized mechanical milling and powder metallurgy techniques. Nanotechnol. Rev..

[31] Kumar R, Jha K, Sharma S, Kumar V, Li C, Eldin EMT (2022). Effect of particle size and weight fraction of SiC on the mechanical, tribological, morphological, and structural properties of Al-5.6Zn-2.2Mg-1.3Cu composites using RSM: fabrication, characterization, and modelling. Heliyon.

[32] Kumar R, Sharma S, Singh JP, Gulati P, Singh G, Dwivedi S (2023). Enhancement in wear-resistance of 30MNCRB5 Boron steel-substrate using HVOF thermal sprayed WC-10% Co 4% Cr coatings: A comprehensive research on microstructural, tribological, and morphological analysis. J. Mater. Res. Technol..

[33] Singh Y (2023). Studies on physical, micro-structural, and slurry erosion behaviour of cold sprayed Ni-20CrþTiCþRe coatings on SA516 steel for high temperature applications. Surf. Rev. Lett..

[34] Phani P (2023). Mechanical properties of carbon fiber reinforced with carbon nanotubes and graphene filled epoxy composites: experimental and numerical investigations. Mater. Res. Express.

[35] Kumar R (2023). Current development of carbide free bainitic and retained austenite on wear resistance in high silicon steel. J. Mater. Res. Technol..

[36] Kiranakumar V (2022). A review on electrical and gas-sensing properties of reduced graphene oxide-metal oxide nanocomposites. Biomass Convers. Biorefin..

[37] Shahid M (2022). A brief assessment on recent developments in efficient electrocatalytic nitrogen reduction with 2D nonmetallic nanomaterials. Nanomaterials.

[38] Singh B (2023). A future prospects and current scenario of aluminium metal matrix composites characteristics. Alex. Eng. J..

[39] Kumar MS, Sathisha N, Manjnatha S (2023). Fatigue surface analysis of AL A356 alloy reinforced hematite metal matrix composites. Biomass Convers. Biorefin..

[40] Bakhtawar S, Muhammad W, Syed G, Umar A, Shafaq M, Niaz K (2022). The impact of laminations on the mechanical strength of carbon fiber composites for prosthetic foot fabrication. Crystals.

[41] Dikshit MK, Singh S, Pathak VK, Saxena KK, Agrawal MK, Malik V (2023). Surface characteristics optimization of biocompatible Ti6Al4V with RCCD and NSGA II using die sinking EDM. J. Mater. Res. Technol..

[42] Lashin MMA, Ibrahim MZ, Khan MI, Guedri K, Saxena KK, Eldin SM (2022). Fuzzy control modeling to optimize the hardness and geometry of laser cladded Fe based MG single track on stainless steel substrate prepared at different surface roughness. Micromachines.

[43] Vemanaboina H, Babu MM, Prerana IC, Gundabattini E, Yelamasetti B, Saxena KK (2023). Evaluation of residual stresses in CO_2_ laser beam welding of SS316L weldments using FEA. Mater. Res. Express.

[44] Ganeshkumar S, Kumar A, Maniraj J, Babu YS, Ansu AK, Goyal A, Kadhim IK, Saxena KK, Prakash C, Altuijri R, Khan MI (2023). Exploring the potential of nano technology: A assessment of nano-scale multi-layered-composite coatings for cutting tool performance. Arab. J. Chem..

[45] Sundaramali G, Jeeva PA, Karthikeyan S, Kandavel TK, Arulmurugan B, Rajkumar S (2023). Experimental investigations of electrodeposited Zn-Ni, Zn-Co, and Ni-CrCo-based novel coatings on AA7075 substrate to ameliorate the mechanical, abrasion, morphological, and corrosion properties for automotive applications. Rev. Adv. Mater. Sci..

[46] Singh G (2022). Impact of post-heat treatment on the surface-roughness, residual stresses, and micromorphology characteristics of plasma-sprayed pure hydroxyapatite and 7%-Aloxite reinforced hydroxyapatite coatings deposited on titanium alloy-based biomedical implants. J. Mater. Res. Technol..

[47] Amer A (2022). Reducing PV soiling and condensation using hydrophobic coating with brush and controllable curtains. Int. J. Low Carbon Technol..

[48] Al-Tameemi HA, Al-Dulaimi T, Awe MO, Sharma S, Pimenov DY (2021). Evaluation of cutting-tool coating on the surface roughness and hole dimensional tolerances during drilling of Al6061-t651 alloy. Materials.

[49] Li H (2022). Extreme pressure and antiwear additives for lubricant: academic insights and Perspectives. Int. J. Adv. Des. Manuf. Technol..

[50] Singh G, Mittal M, Singh J, Sharma S, Gill AS, Chohan JS (2022). Study on the morphological and mechanical properties of TaC reinforced plasma spray coating deposited on titanium alloy. Mater. Today Proc..

[51] Singh G, Mittal M, Singh J, Sharma S, Chohan JS, Kumar R (2022). Effect of post coating processing on the morphological and mechanical properties of plasma Spray reinforced hydroxyapatite coating. Mater. Today Proc..

[52] Sharma S, Xiao ND, Peng W, Chen L (2020). Electrochemical deposited CuNi binary and Cu-Ni-Mn ternary alloys from sulphate bath for anti-corrosive coating applications in Brine environment: Effect of Corrosion behaviour, Polarization studies, Morphological and structural characterizations. Key Eng. Mater..

[53] Singh G, Hitesh V, Amit B, Sachit V, Sharma S (2020). Microwave cladding of Inconel-625 on mild steel substrate for corrosion protection. Mater. Res. Express.

[54] Dwivedi S, Sharma S (2023). Development and characterization of Cu-Ni-Al2O3 surface composite developed by friction stir process technique. Mater. Today Commun..

[55] Dwivedi S, Sharma S (2024). Synthesis of high entropy alloy AlCoCrFeNiCuSn reinforced AlSi7Mg0.3 based composite developed by solid state technique. Mater. Lett..

[56] Ganeshkumar S, Singh BK, Kumar SD, Gokulkumar S, Sharma S, Mausam K (2022). Study of wear, stress and vibration characteristics of silicon carbide tool inserts and nano multi-layered titanium nitride-coated cutting tool inserts in turning of SS304 steels. Materials.

[57] Dwivedi S, Sharma S, Srivastava A, Sethi V, Mohammed K, Kumar A, Khan M, Abbas M, Tag-Eldin E (2023). Homogeneity, metallurgical, mechanical, wear, and corrosion behavior of Ni and B4C coatings deposited on 304 stainless steels developed by microwave cladding technique. J. Mater. Res. Technol..

[58] Sharma S, Dwivedi S, Li C, Awwad F, Khan M, Ismail E (2023). Unveiling of grain structure, porosity, phase distributions, microstructural morphology, surface hardness, and tribo-corrosion characteristics of nickel, and titanium dioxide-based SS-304 steel microwave composite coatings cladding. J. Mater. Res..

[59] Dwivedi S, Sharma S (2023). Metallic cladding through microwave energy of the mixture of Ni and 15% SiC powder on AISI 304: A green approach in surface engineering. Proc. Inst. Mech. Eng..

